# Assessing the 3 pillars of housing for eye and vision health outcomes: A scoping review

**DOI:** 10.1016/j.survophthal.2025.12.008

**Published:** 2025-12-31

**Authors:** Sofia E. Parellada, Kishan Avaiya, Khawla M. Elnour, Mikhayla L. Armstrong, Tiffani R. Spaulding, Kate M. Saylor, Maria A. Woodward, Angela R. Elam, Roshanak Mehdipanah, Paula Anne Newman-Casey, Patrice M. Hicks

**Affiliations:** a University of Miami Miller School of Medicine, Miami, FL, United States; b Georgetown University School of Medicine, Washington, DC, United States; c Howard University College of Medicine, Washington, DC, United States; d University of Chicago Pritzker School of Medicine, Chicago, IL, United States; e Department of Ophthalmology and Visual Science, University of Chicago, Chicago, IL, United States; f Taubman Health Sciences Library, University of Michigan, Ann Arbor, MI, United States; g Institute for Healthcare Policy and Innovation, University of Michigan, Ann Arbor, MI, United States; h Department of Ophthalmology and Visual Sciences, University of Michigan, Ann Arbor, MI, United States; i Department of Health Behavior and Health Education, School of Public Health, University of Michigan, Ann Arbor, MI, United States; j Housing Solutions for Health Equity, University of Michigan, Ann Arbor, MI, United States

**Keywords:** Housing, Social risk factors, Social determinants of health, Vision, Eye

## Abstract

In this scoping review, we examine the implications of 3 pillars (housing conditions and quality, residential consistency, and housing affordability) of healthy housing on vision health outcomes. We examine barriers based on geographical locations of the studies and World Health Organization income levels. We identified 11,190 abstracts, with 10,996 articles retrieved. Sixty-three met inclusion criteria. Among these, housing conditions emerged as the most frequently observed housing pillar associated with adverse vision health outcomes, cited in 62.1 % of the studies. Environmental pollution, particularly indoor air quality and exposure to harmful substances, was the most common condition associated with poor vision outcomes.

## 1. Introduction

Social determinants of health are estimated to account for 80–90 % of modifiable health factors, including those that impact vision health outcomes.^1^ The Neighborhood and Built Environment domain is one of the five domains of Social Determinants of Health (SDoH) as defined by Healthy People 2030.^2^ Factors within the Neighborhood and Built Environment domain that can impact vision health outcomes include pollution, transportation, housing, and neighborhood resources. Challenges in accessing adequate housing can have significant implications on health.^3^ Populations experiencing housing disparities often face disproportionately high rates of chronic diseases, including HIV, diabetes, hypertension, asthma, and increased risk of cancer, anxiety, and depression.^3,4^

Indices such as the Area Deprivation Index and the Distressed Communities Index, which include measures of housing quality, crowding, and affordability, have been associated with worse vision outcomes. Higher levels of neighborhood deprivation have also been independently associated with a greater odds of vision difficulty and blindness, underscoring the importance of examining the housing-related components within these indices.^5^ This is consistent with the Tiebout hypothesis, which proposes that an individual’s income and wealth influence their residential choices and access to various amenities, including healthcare.^6^ Consequently, individuals with limited financial resources may have limited housing options, making it more difficult to receive routine eye care.

The pillars of housing and health include housing affordability, housing conditions and quality, residential consistency, and neighborhood factors. Our study utilized the housing framework by Hernández and Swope to both assess and organize the literature.^7^ The housing conditions and quality pillar examines whether the physical hardware or environmental conditions of a building or unit are adequate. Poor housing conditions have been linked to general health issues, which in turn can affect vision health. Exposure to pollutants has been associated with an increased risk of dry eye disease, glaucoma, and age-related macular degeneration, emphasizing the need to consider environmental factors in housing that may contribute to ocular diseases.^2^ The residential consistency pillar assesses the stability of the residents and whether they can reside in that home for as long as they wish or if they are faced with multiple moves or eviction. People who do not have stable, safe, secure housing have disproportionately high rates of chronic diseases.^8^ For example, according to Maslow’s hierarchy, meeting basic needs like shelter takes priority over seeking preventive healthcare, so individuals facing housing insecurity are less likely to prioritize routine eye care, and traditional clinics may not address their pressing social needs.^9–12^ The housing affordability pillar examines whether a resident can pay their rent or mortgage without financial burden. It is estimated that nearly half of renter households are cost-burdened, as their incomes allocate greater than 30 % of their income to rent each month.^13^ This financial burden diverts financial resources away from health maintenance, which may then negatively impact health outcomes. Finally, the neighborhood factors pillar encompasses the positive or negative health-relevant resources in the surrounding neighborhood. This includes walkability, access to transportation, green spaces and parks for physical activity, and institutions such as grocery stores. Lack of access to transportation can make it difficult for patients to access eye care and limited resources such as grocery stores and green spaces can make it difficult to manage chronic disease such as diabetes, and uncontrolled diabetes can lead to complications including diabetic retinopathy.

This scoping review explored the impact of the pillars of housing on vision health in the United States and internationally. We aim to provide a comprehensive understanding of how housing may impact vision health, which can provide insight into policies and interventions that address housing as a social risk factor impacting eye and vision health outcomes.

## 2. Methods

The proposed search frameworks of Arksey and O’Malley and Levac and colleagues were used for this scoping review, which follows the JBI Manual for Evidence Synthesis: Chapter 11 -Scoping Reviews.^14–16^ A protocol was formulated in advance and reported according to the PRISMA (Preferred Reporting Items for Systematic Reviews and Meta-Analyses) extension for Protocols (PRISMA-P) and is available here: (https://dx.doi.org/10.7302/23639). This review was reported according to the PRISMA extension for scoping review (PRISMA-ScR). The team followed a multi-step, iterative process for developing and refining the search strategy.

### 2.1. Search strategy

The review team collaborated with an informationist (K.M.S) in May, 2023, to identify the target outcomes and created a relevant search strategy, which was utilized to select potential databases to acquire search terms, concepts, and evidence. A study team member (P.M.H) reviewed search terms and results for the databases and provided feedback to obtain the final searches for the databases. EndNote 20 (Clarivate, London, UK) was utilized to manage citations and remove duplicate articles. The databases used for this review include Ovid MEDLINE, Embase (Elsevier), CINAHL Complete (EBSCO), PsycINFO (EBSCO), SocINDEX with Full Text (EBSCO), Web of Science (SCI-EXPANDED, SSCI, and ESCI), and Scopus (Elsevier). The final MEDLINE search strategy can be accessed in Supplemental Figure 1.

### 2.2. Selection of evidence

**Fig. 1.**
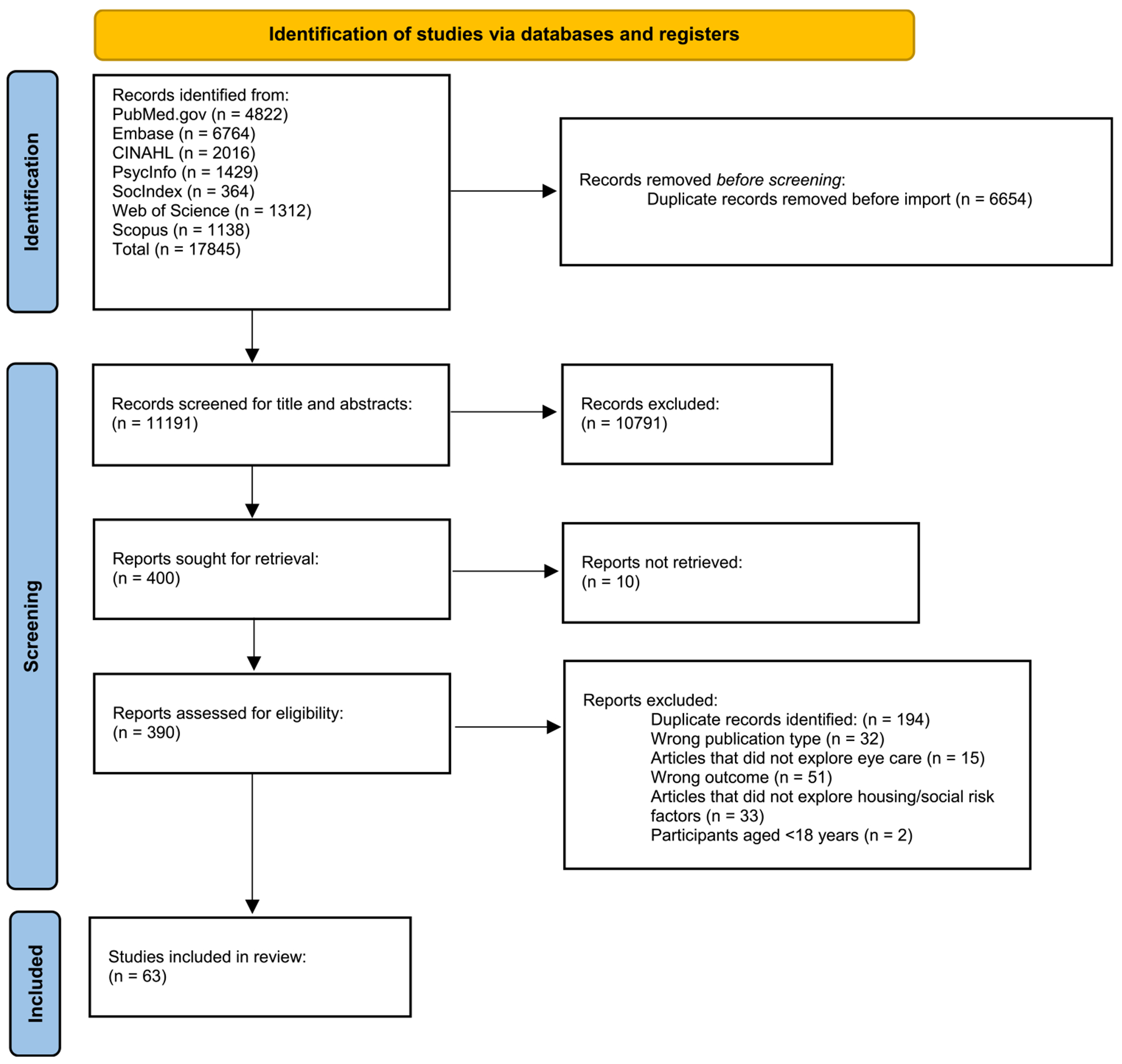
PRISMA flow diagram of study selection for the housing and eye health scoping review.

The Rayyan-Intelligent Systematic Review program (Rayyan Systems Inc., Cambridge, MA) was utilized to review citations. The exclusion criteria were as follows: articles that are reviews, abstracts, case reports, or opinion pieces. We exclusively looked at articles written in English, as we were not able to provide translations of non-English articles. All review screeners completed an initial training by studying the protocol developed for this scoping review. A pilot test was conducted on 10 % of the total articles found, and the screening began once a 75 % agreement was met between two primary screeners for the pilot test (P.M.H. and M. L.A. or S.E.P. or K.E.). During the screening process, at least 2 reviewers analyzed each source at each level (title abstract and full-article review), and disagreements were reconciled by consensus or by a third reviewer. In accordance with the PRISMA-ScR statement, a flowchart and narrative description of the evidence selection process were created and presented in Fig. 1.

#### 2.2.1. Data extraction

Data extracted from the articles included: title, author(s), year of publication, country, aims of the study, location, topic of the study (vision condition or eye care), housing pillar (cost, conditions, consistency) and social risk factor assessed, type of study, and how source findings were obtained (interview, survey, electronic medical record, etc.). Income group by the World Health Organization (WHO) classification system was also identified. Housing pillar information was categorized into explored and observed categorizations, where explored refers to if a study assessed the housing pillar within their study and observed means that the study found the housing pillar was linked to the eye or vision outcome.

#### 2.2.2. Pillars of housing

We analyzed pillars of housing (conditions and quality, residential consistency, affordability) across the sources reviewed to understand if and which of them impact vision health and eye care. The pillars of conditions (housing quality), consistency (residential stability), and cost (housing affordability) come from Swope and Hernández. Reviewers (P. M.H. and M.L.A.) sorted the outcomes of all included studies and categorized them into 1 or more of the 3 pillars.

## 3. Results

### 3.1. Search results and studies included

A search was conducted utilizing the search terms from Supplemental Figure 1. The search yielded a total of 11,190 records. After 194 duplicate records were removed, the remaining 10,996 records were screened by title and abstract. A total of 196 articles were selected for full-text review, of which 133 were excluded for various reasons, including wrong outcome (n = 51), article did not explore housing/social risk factors (n = 33), publication type (32), article did not mention eye care (n = 15), and children under 18 (n = 2). A total of 63 articles (n = 22 in the US, n = 41 international) published between February 2013 and January 2024 were included in this scoping review. A narrative description and flowchart of the evidence selection process is provided in Fig. 1.

The explored and observed pillars of housing for each article by eye condition are found in Supplemental Table 1. Explored and observed risk factors were organized into the 3 pillars of housing according to the codebook outlined in Supplemental Table 2. All articles included and the identified pillars of housing are summarized in Table 1. An explored pillar of housing means that the study looked for correlation between that pillar of housing and an eye condition. If the explored pillar of housing was seen to correlate with an eye condition, it is listed as observed. Multiple eye conditions^5,17–36^(n = 20) had the most number of articles, followed by visual impairment^37–45^ (n = 9), diabetic retinopathy^46–50^ (n = 5), eye irritation^51–54^ (n = 4), dry eye^55–57^ (n = 3), glaucoma^58–60^ (n = 3), trachoma^61–63^ (n = 3), eye infections^64–66^ (n = 3), cataract^67,68^ (n = 2), conjunctivitis^3,29^ (n = 2), eyesight^30,69^ (2 articles), refractive error^49,63^ (n = 2), retinal vein occlusion^70^ (n = 1), neuromyelitis optica spectrum disorder^71^ (n = 1), ocular toxoplasmosis^72^ (n = 1), exfoliation syndrome^73^ (n = 1), and ambylopia^74^ (n = 1). The housing pillars included the following: 2 articles identified affordability, 42 articles identified conditions and quality, and 23 articles identified residential consistency. While studies may have explored more than one pillar of housing, they may have either not observed or only observed a specific pillar of housing in their study. Associations with housing conditions were the most explored (66.7 %) and observed (60.3 %) pillar of housing from the 63 total studies. Affordability was the least explored (3.2 %) and observed (3.2 %) pillar of housing from all the studies. For both US and international studies, conditions were the most explored pillar of housing (59.1 % and 70.7 %, respectively), and affordability was the least explored pillar of housing (9.1 % and 0.0 %, respectively). Of the 59.1 % of US studies that explored conditions, 92.3 % also observed conditions. Of the 70.7 % of international studies that explored conditions, 89.7 % observed conditions. US articles explored all 3 pillars of housing, whereas international articles only explored 2 of the pillars. From the articles explored, US articles observed all 3 pillars of housing: affordability (100.0 %), conditions and quality (92.3 %), and residential consistency (62.5 %). Of the 2 pillars that the international articles explored, they observed conditions (89.7 %) and residential consistency (66.7 %). (Table 2).

Out of 17 different categories of eye conditions studied from the articles included in the review, only “multiple eye conditions” explored all 3 pillars of housing: affordability (10.0 %), conditions and quality (60.0 %), and residential consistency (40.0).^5,17–22,24–36^ This category was defined as articles that researched more than one ophthalmic condition. Of these explored pillars, 100 % of articles observed affordability, 100 % observed conditions, and 62.5 % observed residential consistency. Affordability was only explored and observed by articles that studied multiple eye conditions. Studies that looked at dry eye disease^1,56,57^ (n = 3), glaucoma^58–60^ (n = 3), ocular toxoplasmosis^72^ (n = 1), exfoliation syndrome^73^ (n = 1), conjunctivitis^75,76^ (n = 2), and eyesight^69,77^ (n = 2) only explored conditions, and studies that looked at retinal vein occlusion^70^ (n = 1) and neuromyelitis optica spectrum disorder^71^ (n = 1) only explored consistency. Articles about ocular toxoplasmosis and amblyopia explored correlation between the three pillars of housing but none were observed (Table 3). Studies were also analyzed by income groups based on the World Health Organization (WHO) classification system. Most studies were published or conducted in countries from the High WHO income group (n = 43), with no studies identified from the Low WHO income group. The housing conditions and quality pillar was the most explored across all WHO income groups: High (57.8 %), Upper Middle (87.5 %), and Lower Middle (100.0 %). Of the articles that explored housing conditions and quality, the percentage that also observed conditions was: High (91.3 %), Upper Middle (100 %), and Lower Middle (83.3 %). Interestingly, affordability was only explored and observed by countries that identified in the High WHO income group. (Table 4).

#### 3.1.1. Housing pillars assessed for review

##### 3.1.1.1. Housing conditions and quality.

Factors related to the housing conditions and quality pillar were the most reported association with eye and vision outcomes, observed in 62.1 % of studies (Table 2). All housing conditions and quality measures explored and observed were simplified into 13 categories: pollution, humidity, temperature, home hazards, home age, lighting, location, home type, number of persons residing in the home, home cleanliness, home damage, proper heating and ventilation, and marginal housing. Marginal housing is defined as which is defined as shelters, rooming houses, or single room occupancy hotels, observed a relationship with vision health. Each study that examined and identified an association within these categories was counted accordingly.

A total of 8 studies examined the impact of pollution on eye health, with all reporting a significant effect on vision health.^19,32,52,53,55,56,65,68,77^ Five studies assessed home humidity levels, with 60 % identifying an association between higher humidity in the home and negative ocular symptoms and higher rate of microbial growth in the home.^54,55,57^ Zhang and colleagues controlled for potential confounders, such as age, smoking status, location, and use of air cleaning equipment.^54^ Rock and colleagues controlled for age and sex, but not all environmental factors.^57^ Regarding home temperature, 4 studies explored the influence, but only 1 observed that lower temperatures in the home may be associated with a higher amount of particle surface area on the ocular surface.^56^ Nine studies investigated various home hazards as potential risk factors for vision health, with 4 reporting a significant effect.^17,37,40, 53,63,65^ All 5 studies that examined the role of home lighting found a connection to vision health.^20,34,40,58,59^ Conversely, only 40 % of studies assessing the number of residents in a home observed an effect.^19,46,52,70,77^ No studies reported an association between home damage in the past 12 months and vision health; however, home cleanliness was found to be a contributing factor in 5 out of the 9 studies that considered it.^52–54,57,61,63,64^ Proper heating and ventilation were evaluated in eight studies. In 2 studies, particulate matter (PM) exposure in the home was observed to affect the human ocular surface.^55,56^ Lastly, all studies that examined home type (n = 10) ^17,18,25,26,29,36,44,53,63,73^ and marginal housing (n = 3).^31^

#### 3.1.2. Residential consistency

Residential consistency was reported to have an association with a variety of eye and vision outcomes in 22.7 % of studies (Table 2). Consistency was simplified into 5 categories: number of persons in the home, home ownership, location, length of stay in the home, and housing status (housed vs. unhoused). Of the 5 studies that explored the impact of number of residents in the house, only 40 % found an impact.^67,73^ In a similar fashion, home ownership (renting vs. owned) was explored in eight studies and observed to have an association in 25 % of studies.^67,70^ One study explored location as a factor in housing stability and found it to be influential.^26^ Of the 4 studies assessing the length of stay, 2 identified an association.^21,50,70^ Lastly, housing status was the most frequently reported factor under consistency, with 77 % (10 out of 13 studies) finding a significant link to vision health. ^21,24,27,30,35,39,43,48,66,71,78^

#### 3.1.3. Housing affordability

Cost of housing was the least reported association with eye and vision outcomes, observed in only two studies (3.2 %) (Table 2). Both investigated housing payments and the impact on vision health and found a significant association.^23^ No other cost-related factors were explored or observed (Table 3).

## 4. Discussion

This scoping review mapped the current literature on the impact of housing on eye and vision health utilizing 3 of the 4 pillars of housing as defined by Swope and Hernandez.^76^ We identified 63 studies that explored housing and eye and vision health. In total, 42 studies explored an association between housing conditions and quality and vision health, 23 studies explored an association between residential consistency and vision health, and 2 studies explored housing affordability and vision health. Of these studies, 10 reported an association between housing conditions and quality and vision health, 10 reported an association between residential consistency and vision health, and 2 reported an association between housing affordability and vision health. This is the first review to examine the different dimensions of housing and their associations with eye and vision health outcomes.

Regarding housing conditions and quality, Kaplan and colleagues studied PM in the eye in both clinic and home environments and found that they were associated with select signs of dry eye syndrome (DES).^56^ Specifically, there was a Relationship between PM type, home environment, and DES metrics including tear osmolarity (ρ = −0.60, P = 0.02), inflammation (ρ = 0.53, P = 0.04), and tear break-up time (ρ = 0.56, P = 0.03). Huang and colleagues found similar results, stating that some aspects of DES, such as evoked pain and palpebral conjunctival abnormalities, correlated with PM in the home.^55^ Exposure to PM2.5 may lead to DES through corneal epithelial inflammation and mitochondrial dysfunctions.^79^ Several housing conditions were observed to contribute to symptomatology in patients with eye irritation and infections, including building characteristics (age of building, housing type, and number of persons in the home), home humidity levels, cleanliness, proper heating and ventilation, and pollution.^52,53,61,63–65^ Youssef and colleagues collected data from 73 houses in Morocco on the characteristics of the house and the clinical manifestations of the inhabitants.^53^ They found that there is a statistically significant association between dust in the home and clinical manifestations including eye irritation and blurred vision (Relative Risk = 1.20, 95 % CI:1.05–1.37, p < 0.05). In Brazil, Silva and colleagues found an association between trachoma in students aged 7–16 years old and inadequate living conditions, including unfinished houses i.e., no plastering, painting, flooring, and unfinished bathrooms (OR: 2.27; 95 % CI:1.12–6.48, p = 0.027) and lack of sewage systems (OR: 3.37; 95 % CI: 1.53–7.35, p < 0.001).^63^

It is well recognized that individuals that have unstable housing or who are unhoused have an increased risk of poor health outcomes and hospitalization due to a lack of access to medical care, including but not limited to increased rates of diabetes, myocardial infarctions, cancers, and addiction.^24,80,81^ When looking at residential consistency and visual impairment, a study by Yelle and colleagues examined 95 individuals who were unhoused in 5 different shelters in Montreal.^35^ Ophthalmologists provided examinations and found an age-adjusted 4 times the prevalence of visual impairment in the population (23.6 %), compared to the general Canadian (6 %) population (p < 0.0001). Study participants were also significantly less likely (18.9 % vs. 41.4 %; p < 0.0001) to have had an eye exam in the last year compared to the general population.^35^ Similar trends were observed in other studies, including a study by Noel and colleagues reporting that a population experiencing homelessness in Toronto was found to have an increased prevalence of visual impairment, based on presenting visual impairment with a visual acuity worse than 20/40 in the better eye, compared to the general Canadian population (10.3 % vs 0.5 %; p < 0.001).^27^

The final pillar of housing we examined was housing affordability, examining the association between cost of living and access to eye and vision health. This measure includes expenses such as rent, mortgage, and utilities.^7^ Hom and colleagues based out of the United States, conducted a retrospective cross-sectional study using data from the 2017 National Health Interview Survey to assess worry of housing payments in individuals with eye disease.^23^ Results showed that 17.06 % of individuals with eye conditions reported worrying about housing payments, and individuals that identified as Black race (adjusted odds ratio (aOR): 1.44, 1.16–1.80, p = 0.001) or Hispanic ethnicity (aOR:1.57, 95 % CI: 1.20–2.06, p < 0.001) reported worrying about housing payments more than White individuals. In addition, women (aOR: 1.19, 95 % CI: 1.02–1.38, p = 0.023) were more likely to worry about housing payments compared to men. Individuals living at 100–200 % of the federal poverty line (aOR:2.06; 95 % CI: 1.70–2.49) or below the poverty line (aOR:1.96, 95 % CI: 1.54–2.49, <0.001) were also more likely to report worry about housing payments.^23^ French and colleagues reported that Medicare ocular hospitalizations were greater in communities where severe housing problems were present, including housing costs over 50 % of household monthly income (OR:1.13, 95 % CI: 1.09–1.18, p < 0.01).^19^ Housing cost, as well as housing operating costs, can directly impact eye and vision outcomes by diverting financial resources that could otherwise be allocated to essential health services such as eye examinations and follow-up appointments, treatments including glasses or glaucoma medication, or transportation to access appointments or treatment.^7^ Recent work by Ige and colleagues demonstrates that financial strain can directly impact the quality of eye care, as glaucoma patients in the lowest wealth quartile were significantly less likely to achieve the US National Quality Forum’s recommended intraocular pressure reduction targets and were more likely to be lost to follow-up. These findings reinforce that economic hardship can impede sustained engagement with crucial eye care services.^82^

Of the 63 articles, 41 of them were conducted internationally. The most represented countries were England, India, and Canada. Many of these articles focused on multiple eye conditions (34.1 %), closely followed by issues in visual impairment (14.6 %), eye irritation (7.3 %), and trachoma (7.3 %). The most observed pillar in the international articles was housing conditions and quality with 68.2 % of articles reporting an observed condition. In total, 24.4 % observed problems with residential consistency. None examined housing affordability in their studies. The most observed conditions for international studies included home cleanliness, indoor air pollution, proper heating and ventilation, and home type. The 3 articles that studied trachoma, the leading cause of preventable blindness in the world, were from Brazil, Ghana, and Tanzania. It is well known that the prevalence of trachoma is higher in low-resourced countries.^12^ While Brazil is generally classified as an upper-middle-income country, several regions—particularly in the North, where the study was conducted—face significant unmet needs in healthcare services, access to medications, and poor sanitation.^63,83^ In Tanzania, Chen and colleagues conducted a household survey in the Kongwa region focused on facial cleanliness, a protective factor against trachoma. They found that the household cleanliness index directly correlates with levels of infection.^61^ Specifically, a 0.5-point increase in the community average household cleanliness score was linked to a 2.28 times greater likelihood of reducing trachoma prevalence by one category (i.e., from ≥10 % to 5–9.9 %, or from 5 to 9.9 % to <5 %; odds ratio = 2.28, 95 % CI = 1.17–4.80).

This scoping review identified several gaps in eye and vision research related to the housing pillars. Almost a third of the studies included in the review assessed multiple eye conditions; thus, it is important to examine individual housing pillars associated with each eye condition to draw more precise conclusions about their unique impacts. No studies assessing housing pillars in relation to eye and vision health outcomes were conducted in WHO-designated low-income countries, indicating a need for research in these areas. In the conditions pillar of housing, the air pollutants studied included both Particulate Matter 2.5 and 10. Future research should assess additional air pollutants, such as indoor air pollutants like wood smoke, mold spores, cooking fuels, that have yet to be examined for associations with eye disease and vision health. For the consistency pillar, the factors considered included length of stay in the home, number of people in the home, home ownership, and location of the home. A key measure that was not directly assessed in any of the studies was the impact of short-term housing situations, such as involuntary removal from one’s home and community (i.e., natural disasters, eviction, gentrification, or foreclosure), and their hindrance to eye and vision health-related resources, including healthcare, employment, and social support.^7^ For the affordability pillar, no international studies were conducted; thus, there is a significant need to assess the implications of this housing pillar on eye care utilization and vision health outcomes in diverse settings. Furthermore, the only studies conducted for this pillar were in a high-income country (the US), which do show that lower cost burden improves vision outcomes. As this review has highlighted, the housing pillars have implications for both eye and vision outcomes, so future research is needed in these understudied areas.

There are limitations to this review. First, the results are subject to the limitations of a scoping review which include selection bias, date limitations, and database selection. In addition to this, 41 of the 63 studies included were conducted internationally, limiting the generalizability to the United States. Finally, the pillar of neighborhood factors was not assessed, as that focuses on the residential area and built environment context and not on the home itself.

Housing is a social determinant of health that can contribute to vision health disparities. This review highlights the potential impact that the pillars of housing can have on eye and vision health. It is important to note that in the context of eye care and vision research, each of these pillars has largely been explored in isolation, despite evidence that they influence one another, thus it is important to consider that the impact of a single housing factor on eye and vision health outcomes could also shaped by other related social and housing factors.^7^ For example, if a patient with diabetes utilizes the majority of their income to pay for housing, they may be unable to afford nutritious foods, their medication, and routine care for diabetes management which can lead to unmanaged diabetes and greater risk for diabetic retinopathy.^84^ It could be helpful for eye care clinics to implement a social risk factor screening to account for not only food insecurity, transportation, and medication affordability but also to identify housing instability and quality. Patients that have housing needs can be connected with social workers who can aid in facilitating referrals to rent assistance programs, housing quality improvement programs, and independent living services. At the systems level, developing health policy solutions to address housing insecurity are vital for improving general health and eye and vision health in the United States.

## 5. Conclusion

The housing pillars conditions and quality were reported as most associated with eye and vision outcome. The housing affordability and its association to eye and vision health was the least studied. As the majority of existing studies were conducted internationally, additional research is needed in the United States on the impacts of housing on eye and vision health outcomes. Overall, pillars of health of housing are key social risk factors impacting eye and vision health outcomes, which should be considered when addressing eye and vision health disparities.

### Methods of literature search

This scoping review included peer-reviewed qualitative, quantitative, or mixed-methods studies written in English that examined housing in relation to eye care and vision health among adults aged 18 years and older. We excluded reviews, abstracts, case reports, opinion pieces, and non-English articles due to translation limitations. The full study protocol including methods of literature search has been deposited: https://dx.doi.org/10.7302/23639.

## Supplementary Material

1

2

3

4

## Figures and Tables

**Table 1:** Explored and observed pillars of housing by eye condition.

Author / Year	Country	Explored Pillars of Housing			Pillars of Housing Observed			WHO income group
		Cost	Conditions	Consistency	Cost	Conditions	Consistency	
Dry Eye								
Kaplan *et al.* 2019	USA		X			X		High
Huang *et al.* 2021	USA		X			X		High
Rock *et al.* 2022	USA		X			X		High
Glaucoma								
Bhorade *et al.* 2013	USA		X			X		High
Ramulu *et al.* 2022	USA		X			X		High
Yonge *et al.* 2018	USA		X					High
Cataract								
Vashist *et al.* 2020	India		X			X		Lower middle
Quintana *et al.* 2013	Spain			X			X	High
Diabetic Retinopathy								
Thomas *et al.* 2021	UK			X			X	High
Chauhan *et al.* 2020	India		X			X		Lower middle
Silverberg *et al.* 2021	USA			X				High
Davis *et al.* 2017	USA			X			X	High
Cai *et al.* 2021	USA		X			X		High
Retinal Vein Occlusion								
McDermott *et al.* 2022	USA			X			X	High
Neuromyelitis Optica Spectrum Disorder									
Rafiee *et al.* 2020	Iran			X			X	Upper middle
Ocular Toxoplasmosis								
Abu *et al.* 2016	Ghana		X					Lower middle
Exfoliation Syndrome								
Aygun *et al.* 2023	Turkey		X			X		Upper middle
Amblyopia								
Bountziouka *et al.* 2021	UK			X				High
Conjunctivitis								
Reboux *et al.* 2018	France		X					High
Suryani *et al.* 2021	Indonesia		X			X		Upper middle
Trachoma								
Debrah *et al.* 2017	Ghana		X					Lower middle
Chen *et al.* 2021	Tanzania		X			X		Lower middle
Silva *et al.* 2020	Brazil		X	X		X		Upper middle
Eye Irritation								
Youssef *et al.* 2021	Morocco		X			X		Lower middle
Zhang *et al.* 2018	China		X			X		Upper middle
Johnston *et al.* 2022	USA		X			X		High
Yang *et al.* 2021	Sweden		X	X		X		High
Eye Infections								
Firdaus *et al.* 2012	India		X			X		Lower middle
Lopez *et al.* 2024	USA			X			X	High
Carnt *et al.* 2020	UK		X			X		High
Eyesight								
Melody *et al.* 2016	Australia		X			X		High
Shiue *et al.* 2015	USA		X			X		High
Visual Impairment								
Lee *et al.* 2018	Korea		X			X		High
Brown *et al.* 2016	USA			X			X	High
Lebrun-Harris *et al.* 2012	USA			X				High
Pesonen *et al.* 2022	Finland		X			X		High
Tham *et al.* 2018	Singapore		X			X		High
Pooprasert *et al.* 2020	UK			X			X	High
Alvarado-Esquivel *et al.* 2015	Mexico		X			X		Upper middle
Wong *et al.* 2020	Hong Kong			X			X	High
Andersson *et al.* 2020	USA		X			X		High
Refractive Error								
D’ath *et al.* 2016	UK			X			X	High
Marmamula *et al.* 2020	India		X			X		Lower middle
Multiple Eye Conditions								
Haanes *et al.* 2015	Norway		X			X		High
Hom *et al.* 2021	USA	X			X			High
Hennein *et al.* 2021	USA			X			X	High
French *et al.* 2019	USA	X	X		X	X		High
Slomovic *et al.* 2023	Canada		X			X		High
Abdu *et al.* 2013	Nigeria		X	X		X		Lower middle
Wang *et al.* 2023	China		X			X		Upper middle
Sutradhar *et al.* 2019	Bangladesh		X			X		Lower middle
Hennein *et al.* 2020	USA			X				High
Sukhsohale *et al.* 2013	India		X			X		Lower middle
Elliott *et al.* 2019	USA		X			X		High
Park *et al.* 2024	Canada			X				High
Jiang *et al.* 2020	Canada			X			X	High
Low *et al.* 2020	Singapore		X			X		High
Yelle *et al.* 2022	Canada			X			X	High
Sawers *et al.* 2016	UK			X			X	High
Marmamula *et al.* 2023	India		X			X		Lower middle
Yekta *et al.* 2019	Iran		X			X		Upper middle
Noel *et al.* 2015	Canada			X			X	High
Ryu *et al.* 2018	USA		X			X		High

Notes: “X” indicates that the pillar was explored or observed in the study. WHO = World Health Organization.

**Table 2:** Pillars of housing and percentage by location.

Pillars of Housing	Explored Pillars of Housing			Pillars of Housing Observed		
	Total (n = 63)	United States (n = 22)	International (n = 41)	Total (n = 63)	United States (n = 22)	International (n = 41)
Cost	3.2 % (2)	9.1 % (2)	0.0 % (0)	3.2% (2)	100.0 % (2)	0.0 % (0)
Conditions	66.7 % (42)	59.1% (13)	70.7 % (29)	60.3 % (38)	92.3% (12)	89.7 % (26)
Consistency	36.5 % (23)	36.4 % (8)	36.6 % (15)	23.8 % (15)	62.5 % (5)	66.7 % (10)

**Table 3:** Pillars of housing and percentage by eye condition.

	Explored Pillars of Housing			Pillars of Housing Observed		
Condition	Cost	Conditions	Consistency	Cost	Conditions	Consistency
Dry eye disease (3)	0.0 % (0)	100 % (3)	0.0 % (0)	0.0 % (0)	100.0 % (3)	0.0 % (0)
Glaucoma (3)	0.0 % (0)	100 % (3)	0.0 % (0)	0.0 % (0)	66.7 % (2)	0.0 % (0)
Cataract (2)	0.0 % (0)	50 % (1)	50 % (1)	0.0 % (0)	100.0% (1)	100.0 % (1)
Diabetic Retinopathy (5)	0.0 % (0)	40.0 % (2)	60.0 % (3)	0.0 % (0)	100.0 % (2)	66.7 % (2)
Retinal Vein Occlusion (1)	0.0 % (0)	0.0 % (0)	100.0 % (1)	0.0 % (0)	0.0 % (0)	100.0 % (1)
Neuromyelitis Optica Spectrum Disorder (1)	0.0 % (0)	0.0 % (0)	100.0 % (1)	0.0 % (0)	0.0 % (0)	100.0 % (1)
Ocular Toxoplasmosis (1)	0.0 % (0)	100.0% (1)	0.0 % (0)	0.0 % (0)	0.0 % (0)	0.0 % (0)
Exfoliation Syndrome (1)	0.0 % (0)	100.0% (1)	0.0 % (0)	0.0 % (0)	100.0% (1)	0.0 % (0)
Amblyopia (1)	0.0 % (0)	0.0 % (0)	100.0 % (1)	0.0 % (0)	0.0 % (0)	0.0 % (0)
Conjunctivitis (2)	0.0 % (0)	100.0 % (2)	0.0 % (0)	0.0 % (0)	50.0 % (1)	0.0 % (0)
Trachoma (3)	0.0 % (0)	100.0 % (3)	33.3 % (1)	0.0 % (0)	66.7 % (2)	0.0 % (0)
Eye Irritation (4)	0.0 % (0)	100.0 % (4)	25.0 % (1)	0.0 % (0)	100.0 % (4)	0.0 % (0)
Eye Infections (3)	0.0 % (0)	66.7 % (2)	33.3 % (1)	0.0 % (0)	100.0 % (2)	100.0 % (1)
Eyesight (2)	0.0 % (0)	100.0 % (2)	0.0 % (0)	0.0 % (0)	100.0 % (2)	0.0 % (0)
Visual Impairment (9)	0.0 % (0)	55.6 % (5)	44.4 % (4)	0.0 % (0)	100.0 % (5)	75.0 % (3)
Refractive Error (2)	0.0 % (0)	50 % (1)	50 % (1)	0.0 % (0)	100.0% (1)	100.0 % (1)
Multiple Eye Conditions (20)	10.0 % (2)	60.0% (12)	40.0 % (8)	100.0 % (2)	100.0% (12)	62.5 % (5)

**Table 4:** Pillars of housing and percentage and frequency by WHO income groups.

	Explored Pillars of Housing					Pillars of Housing Observed				
		WHO income group					WHO income group			
Pillars of Housing	Total (n = 63)	High (n = 43)	Upper Middle (n = 8)	Lower Middle (n = 12)	Low (n = 0)	Total (n = 63)	High (n = 43)	Upper Middle (n = 8)	Lower Middle (n = 12)	Low (n = 0)
Cost	3.2 % (2)	4.7 % (2)	0.0 % (0)	0.0 % (0)	0.0 % (0)	3.2% (2)	100.0 % (2)	0.0 % (0)	0.0 % (0)	0.0 % (0)
Conditions	66.7 % (42)	57.8 % (23)	87.5 % (7)	100.0 %(12)	0.0 % (0)	60.3 % (38)	91.3 % (21)	100.0 % (7)	83.3 % (10)	0.0 % (0)
Consistency	36.5 % (23)	46.5 % (20)	25.0 % (2)	8.3 %(1)	0.0 % (0)	23.8 % (15)	70.0 % (14)	50.0 % (1)	0.0 % (0)	0.0 % (0)

## Appendix A. Supporting information

Supplementary data associated with this article can be found in the online version at doi:10.1016/j.survophthal.2025.12.008.

## References

[1] Hood CM, Gennuso KP, Swain GR, Catlin BB. County health rankings: relationships between determinant factors and health outcomes. Am J Prev Med. 2016;50(2): 129–135. 26526164 10.1016/j.amepre.2015.08.024

[2] Gomez CA, Kleinman DV, Pronk N, Addressing health equity and social determinants of health through healthy people 2030. J Public Health Manag Pract. 2021;27(6):S249–S257. 10.1097/PHH.0000000000001297. 33729197 PMC8478299

[3] Rolfe S, Garnham L, Godwin J, Housing as a social determinant of health and wellbeing: developing an empirically-informed realist theoretical framework. BMC Public Health. 2020;20:1138. 10.1186/s12889-020-09224-0. 32689966 PMC7370492

[4] Lee JJ, Jagasia E, Wilson PR. Addressing health disparities of individuals experiencing homelessness in the U.S. with community institutional partnerships: an integrative review. J Adv Nurs. 2023;79(5):1678–1690. 10.1111/jan.15591. 36882981 PMC10182242

[5] Hicks PM, Lin G, Newman-Casey PA, Place-based measures of inequity and vision difficulty and blindness. JAMA Ophthalmol. 2024;142(6). 10.1001/jamaophthalmol.2024.1207. PMC11082749 38722650

[6] Tiebout CM. A pure theory of local expenditures. J Polit Econ. 1956;64(5):416–424.

[7] Swope CB, Hernández D Housing as a determinant of health equity: a conceptual model. Soc Sci amp Med. 2020:243. 10.1016/j.socscimed.2019.112571. PMC7146083 31675514

[8] Garg A, Boynton-Jarrett R, Dworkin PH. Avoiding the unintended consequences of screening for social determinants of health. JAMA. 2016;316(8):813–814. 10.1001/jama.2016.9282. 27367226

[9] Gelberg L, Gallagher TC, Andersen RM, Koegel P. Competing priorities as a barrier to medical care among homeless adults in Los Angeles. Am J Public Health. 1997;87(2):217–220. 10.2105/ajph.87.2.217. 9103100 PMC1380797

[10] Kushel MB, Gupta R, Gee L, Haas JS. Housing instability and food insecurity as barriers to health care among low-income Americans. J Gen Intern Med. 2006;21(1):71–77. 10.1111/j.1525-1497.2005.00278.x. 16423128 PMC1484604

[11] Maslow AH Motivation and personality. 1954;411. Available at: 〈https://psycnet.apa.org/fulltext/1955-02233-000.pdf〉.

[12] Solomon AW, Burton MJ, Gower EW, Trachoma. Published 2022 May 26. Nat Rev Dis Prim. 2022;8(1):32. 10.1038/s41572-022-00359-5 35618795

[13] Census Bureau US. Nearly half of renter households are cost-burdened, proportions differ by race. U S Dep Commer; 2024. 〈https://www.census.gov/newsroom/press-releases/2024/renter-households-cost-burdened-race.html〉.

[14] Arksey H Scoping studies: towards a methodological framework. Int J Soc Res Method. 2005;8(1):19–32.

[15] Levac D, Colquhoun H. Scoping studies: advancing the methodology. Implement Sci. 2010;5:69. 20854677 10.1186/1748-5908-5-69 PMC2954944

[16] Peters MDJ, Godfrey C, McInerney P, Chapter 11: scoping reviews (2020 version). In: Aromataris E, Munn Z, eds. JBI manual for evidence synthesis. JBI; 2020. 〈https://synthesismanual.jbi.global〉.

[17] Abdu L, Withers J, Habib AG, Mijinyawa MS, Yusef SM. Disease pattern and social needs of street people in the race course area of Kano, Nigeria. hpu. 2013;24(1):97. 10.1353/hpu.2013.0010. 23377721

[18] Elliott AF, Heskett M, Spiker C, Mcgwin G, Owsley C. Low rates of eye care utilization among visually impaired subsidized senior housing residents. Aging Ment Health. 2019;25(2):360. 10.1080/13607863.2019.1683813. 31694391 PMC7202945

[19] French DD, Wang A, Prager AJ, Margo CE. Association of the robert wood johnson foundations’ social determinants of health and medicare ocular hospitalizations: a cross sectional data analysis. Ophthalmol Ther. 2019;8(4):611. 10.1007/s40123-019-00220-1. 31677061 PMC6858415

[20] Haanes G, Kirkevold M, Hofoss D, Horgen G, Eilertsen G. An intervention designed to improve sensory impairments in the elderly and indoor lighting in their homes: an exploratory randomized controlled trial. JMDH. 2015. 10.2147/jmdh.s71718. PMC4295531 25678795

[21] Hennein L, De Alba Campomanes AG. Association of a health coaching and transportation assistance intervention at a free ophthalmology homeless shelter clinic with follow-up rates. JAMA Ophthalmol. 2021;139(3). 10.1001/jamaophthalmol.2020.6373. PMC7844689 33507215

[22] Hennein L, Spaulding KA, Karlegan V, Nnamani Silva ON, De Alba Campomanes AG. Follow-up rates at a free ophthalmology clinic at a homeless shelter. J Acad Ophthalmol. 2020;13(01), e51. 10.1055/s-0041-1726288. PMC9927987 37389160

[23] Hom GL, Cwalina TB, Jella TK, Singh RP. Assessing financial insecurity among common eye conditions: a 2016-2017 national health survey study. Eye. 2021;36(10):2044. 10.1038/s41433-021-01745-1. 34426657 PMC8380859

[24] Hwang SW. Homelessness and health. CMAJ. 2001;164(2):229–233. 11332321 PMC80688

[25] Low JR, Gan ATL, Fenwick EK, Role of socio-economic factors in visual impairment and progression of diabetic retinopathy. Br J Ophthalmol. 2020;105(3):420. 10.1136/bjophthalmol-2020-316430. 32430341

[26] Marmamula S, Barrenkala NR, Kumbham TR, Brahmanandam Modepalli S, Keeffe J. Unilateral vision loss in elderly people in residential care: prevalence, causes and impact on visual functioning: The hyderabad ocular morbidity in elderly study (HOMES). Ophthalmic Epidemiol. 2023;30(3):260. 10.1080/09286586.2022.2104323. 35892240 PMC7615316

[27] Noel CW, Fung H, Srivastava R, Visual impairment and unmet eye care needs among homeless adults in a canadian city. JAMA Ophthalmol. 2015;133(4). 10.1001/jamaophthalmol.2014.6113. 25654733

[28] Park T, Issa M, Mikhail M, Ophthalmic findings in marginally housed women in a canadian city. Can J Ophthalmol. 2024;59(1):12. 10.1016/j.jcjo.2022.11.005. 36442515

[29] Ryu E, Olson JE, Juhn YJ, Association between an individual housing-based socioeconomic index and inconsistent self-reporting of health conditions: a prospective cohort study in the mayo clinic biobank. BMJ Open. 2018;8(5). 10.1136/bmjopen-2017-020054. PMC5961601 29764878

[30] Sawers N The state of ocular health among london’s homeless population. Eye. 2016;31(4):632. 10.1038/eye.2016.283. 28009348 PMC5396003

[31] Slomovic J, Hanna V, Chaban Y, Delivering eye care to homeless and marginally housed populations during the COVID-19 pandemic: a pilot study. Can J Ophthalmol. 2023;58(2):136. 10.1016/j.jcjo.2021.08.018. 34563495 PMC8418907

[32] Sukhsohale ND, Narlawar UW, Phatak MS. Indoor air pollution from biomass combustion and its adverse health effects in central india: an exposure-response study. Indian J Community Med. 2013;38(3). 10.4103/0970-0218.116353. PMC3760325 24019602

[33] Sutradhar I, Gayen P, Hasan M, Gupta RD, Roy T, Sarker M. Eye diseases: the neglected health condition among urban slum population of Dhaka, Bangladesh. BMC Ophthalmol. 2019;19(1). 10.1186/s12886-019-1043-z. PMC6357461 30704423

[34] Wang C, Leung M. Effects of subjective perceptions of indoor visual environment on visual-related physical health of older people in residential care homes. Build Environ. 2023;237. 10.1016/j.buildenv.2023.110301.

[35] Yelle B, Beaulieu K, Etty MC, The prevalence and causes of visual impairment among the male homeless population of Montreal, Canada. Clin Exp Optom. 2022; 106(4):431–435. 10.1080/08164622.2022.2036578. 35156540

[36] Yekta R, Hashemi H, Pakzad R, Visual impairment and some of ocular problem in nursing home residents. Br J Vis Impair. 2019;37(3):194–204. 10.1177/0264619619839754.

[37] Alvarado-Esquivel C, Hernandez-Tinoco J, Sanchez-Anguiano LF. Seroepidemiology of toxocara infection in patients with vision impairment and blindness in durango, mexico. J Clin Med Res. 2015;7(3):176. 10.14740/jocmr2032w. 25584103 PMC4285064

[38] Andersson RBÅ, Al-Namaeh M, Monaco WA, Meng H. Vision loss among delaware nursing home residents. Gerontol Geriatr Med. 2020;6. 10.1177/2333721420934245. PMC7313322 32637462

[39] Brown RT, Hemati K, Riley ED, Geriatric conditions in a population-based sample of older homeless adults. GERONT. 2016. 10.1093/geront/gnw011. PMC5881727 26920935

[40] Lee SY, Yoo SE. Effects of housing conditions and environmental factors on accidents and modification intention of the vision impaired. J Asian Archit Build Eng. 2018;14(2):347. 10.3130/jaabe.14.347.

[41] Lebrun-harris LA, Baggett TP, Jenkins DM, Health status and health care experiences among homeless patients in federally supported health centers: findings from the 2009 patient survey. Health Serv Res. 2012;48(3):992. 10.1111/1475-6773.12009. 23134588 PMC3681240

[42] Pesonen T, Saarela K, Falck A, Edgren J, Kyngäs H, Siira H. Visual impairment and the need for vision care services amongst older finnish people receiving home care. Nurs Open. 2022;10(4):2519. 10.1002/nop2.1510. 36564916 PMC10006660

[43] Pooprasert P, Ahnood D, Parmar T, Wang W, Young-Zvandasara T, Morgan J. Prevalence of refractive error, visual impairment and access to eyecare for the homeless in wales, united kingdom. Eye. 2020;35(10):2727. 10.1038/s41433-020-01271-6. 33235337 PMC8452665

[44] Tham Y, Lim S, Shi Y, Trends of visual impairment and blindness in the Singapore chinese population over a decade. Sci Rep. 2018;8(1). 10.1038/s41598-018-30004-9. PMC6093864 30111785

[45] Wong PW, Lau JK, Choy BN, Sociodemographic, behavioral, and medical risk factors associated with visual impairment among older adults: a community-based pilot survey in southern district of hong kong. BMC Ophthalmol. 2020;20(1). 10.1186/s12886-020-01644-1. PMC7501719 32948134

[46] Cai CX, Li Y, Zeger SL, Mccarthy ML. Social determinants of health impacting adherence to diabetic retinopathy examinations. BMJ Open Diab Res Care. 2021;9(1). 10.1136/bmjdrc-2021-002374. PMC8479983 34583972

[47] Chauhan A, Verma AK, Sharma D, Difference in prevalence of diabetes and diabetic retinopathy among low-altitude dwellers vs. high-altitude dwellers in North India. Asian J Ophthalmol. 2020;17(1):19–29. 10.35119/asjoo.v17i1.441.

[48] Davis JA, Tsui I, Gelberg L, Gabrielian S, Lee ML, Chang ET. Risk factors for diabetic retinopathy among homeless veterans. Psychol Serv. 2017;14(2):221–228. 10.1037/ser0000148. 28481608

[49] Silverberg EL, Sterling TW, Williams TH, Castro G, Rodriguez De La Vega P, Barengo NC. The association between social determinants of health and self-reported diabetic retinopathy: an exploratory analysis. IJERPH. 2021;18(2). 10.3390/ijerph18020792. PMC7832397 33477729

[50] Thomas RL, Cheung W, Rafferty JM, Luzio SD, Akbari A, Owens DR. Characteristics of repeat non-attenders at diabetes eye screening wales, a national community-based diabetes-related retinopathy screening service, during 2003–2018. Diabet Med. 2021;38(9). 10.1111/dme.14536. 33545742

[51] Jiang S, Mikhail M, Slomovic J, Prevalence and impact of eye disease in an urban homeless and marginally housed population. Can J Ophthalmol. 2020;55(1): 76. 10.1016/j.jcjo.2019.07.006. 31712023

[52] Yang Q, Wang J, Norbäck D The home environment in a nationwide sample of multi-family buildings in Sweden: associations with ocular, nasal, throat and dermal symptoms, headache, and fatigue among adults. Indoor Air. 2021;31(5):1402. 10.1111/ina.12787. 33682978

[53] Youssef B, Belkacem K, Abderrahmane A, Association between housing characteristics and population health. Ann Clin Anal Med. 2021;12(_03):290. 10.4328/acam.20528.

[54] Zhang X, Norbäck D, Fan Q, Dampness and mold in homes across china: associations with rhinitis, ocular, throat and dermal symptoms, headache and fatigue among adults. Indoor Air. 2018;29(1):30. 10.1111/ina.12517. 30379348

[55] Huang A, Janecki J, Galor A, Association of the indoor environment with dry eye metrics. JAMA Ophthalmol. 2021;138(8). 10.1001/jamaophthalmol.2020.2237. PMC7333174 32614410

[56] Kaplan C, Galor A, Blackwelder P, Human ocular surface particulate composition in the clinical versus home environment. Cornea. 2019;38(10):1266–1272. 10.1097/ico.0000000000002087. 31356416

[57] Rock S, Galor A, Kumar N. Indoor airborne microbial concentration and dry eye. Am J Ophthalmol. 2022;223:193. 10.1016/j.ajo.2020.10.003. PMC9580028 33065065

[58] Bhorade AM, Perlmutter MS, Wilson B, Differences in vision between clinic and home and the effect of lighting in older adults with and without glaucoma. JAMA Ophthalmol. 2013;131(12). 10.1001/jamaophthalmol.2013.4995. PMC4377300 24263699

[59] Ramulu PY, Mihailovic A, Miller RB, West SK, Gitlin LN, Friedman DS. Environmental features contributing to falls in persons with vision impairment: the role of home lighting and home hazards. Am J Ophthalmol. 2022;230:207. 10.1016/j.ajo.2021.04.022. PMC8560652 33951447

[60] Yonge AV, Swenor BK, Miller R, Quantifying fall-related hazards in the homes of persons with glaucoma. Ophthalmology. 2018;124(4):562. 10.1016/j.ophtha.2016.11.032. PMC5365348 28017422

[61] Chen X, Munoz B, Wolle MA, Environmental factors and hygiene behaviors associated with facial cleanliness and trachoma in Kongwa, Tanzania. PLoS Negl Trop Dis. 2021;15(10). 10.1371/journal.pntd.0009902. PMC8577779 34710082

[62] Debrah O, Mensah EO, Senyonjo L, Elimination of trachoma as a public health problem in Ghana: providing evidence through a pre-validation survey. PLoS Negl Trop Dis. 2017;11(12). 10.1371/journal.pntd.0006099. PMC5746280 29232708

[63] Silva EJD, Pereira DP, Ambrózio JOAM, Barboza LM, Fonseca VL, Caldeira AP. Prevalence of trachoma and associated factors in students from the Jequitinhonha Valley, Minas Gerais, Brazil. Rev Soc Bras Med Trop. 2020;53. 10.1590/0037-8682-0056-2020. PMC7580275 33111907

[64] Carnt NA, Subedi D, Connor S, Kilvington S. The relationship between environmental sources and the susceptibility of acanthamoeba keratitis in the United Kingdom. PLoS ONE. 2020;15(3). 10.1371/journal.pone.0229681. PMC7065798 32160218

[65] Firdaus G, Ahmad A. Relationship between housing and health: a cross-sectional study of an urban centre of India. Indoor Built Environ. 2012;22(3):498–507. 10.1177/1420326x12443846.

[66] Lopez JB, Chan L, Saifee M, Padmanabhan S, Yung M, Chan MF. Risk factors predicting loss to follow-up, medication noncompliance, and poor visual outcomes among patients with infectious keratitis at a public county hospital. Cornea. 2024;42(9):1069. 10.1097/ico.0000000000003121. PMC9958267 36036690

[67] Quintana JM, Garcia S, Aguirre U, Relationship of sociodemographic variables with outcomes after cataract surgery. Eye. 2013;27(6):698. 10.1038/eye.2013.85. 23703627 PMC3682372

[68] Vashist P, Tandon R, Murthy GVS, Association of cataract and sun exposure in geographically diverse populations of india: the CASE study. First report of the ICMR-EYE SEE study group. PLoS ONE. 2020;15(1). 10.1371/journal.pone.0227868. PMC6977762 31971985

[69] Shiue I Indoor mildew odour in old housing was associated with adult allergic symptoms, asthma, chronic bronchitis, vision, sleep and self-rated health: USA NHANES, 2005–2006. Environ Sci Pollut Res. 2015;22(18):14234. 10.1007/s11356-015-4671-8. 25971810

[70] Mcdermott JJ, Lee TC, Chan AX, Novel association between opioid use and increased risk of retinal vein occlusion using the national institutes of health all of us research program. Ophthalmol Sci. 2022;2(1). 10.1016/j.xops.2021.100099. PMC9205363 35721456

[71] Rafiee F, Tarjoman T, Moghadasi AN, Stressful life events, socioeconomic status, and the risk of neuromyelitis optica spectrum disorder: a population-based case-control study. Mult Scler Relat Disord. 2020;46. 10.1016/j.msard.2020.102544. 33032056

[72] Abu EK, Boampong JN, Amoabeng JK, Epidemiology of ocular toxoplasmosis in three community surveys in the central region of Ghana, West Africa. Ophthalmic Epidemiol. 2016;23(1):14–19. 10.3109/09286586.2015.1089579. 26786055

[73] Aygun FB, Bosi ATB, Kocabeyoglu S, Irkec M. Evaluation of the effects of environmental factors and eating habits on exfoliation syndrome and glaucoma in a Turkish population. Eur J Ophthalmol. 2023;34(1):168–174. 10.1177/11206721231178055. 37226472

[74] Bountziouka V, Cumberland PM, Rahi JS. Impact of persisting amblyopia on socioeconomic, health, and well-being outcomes in adult life: findings from the UK biobank. Value Health. 2021;24(11):1603. 10.1016/j.jval.2021.05.010. 34711360

[75] Reboux G, Rocchi S, Laboissière A, Survey of 1012 moldy dwellings by culture fungal analysis: Threshold proposal for asthmatic patient management. Indoor Air. 2018;29(1):5. 10.1111/ina.12516. 30368912

[76] Suryani L, Setyandriana Y, Meida NS. The social-environmental risk factor for conjunctivitis. Open Access Maced J Med Sci. 2021;9(T4):319. 10.3889/oamjms.2021.5787.

[77] Melody SM, Bennett E, Clifford HD, A cross-sectional survey of environmental health in remote Aboriginal communities in Western Australia. Int J Environ Health Res. 2016;26(5–6):525–535. 10.1080/09603123.2016.1194384. 27267619

[78] D’ath PJ, Keywood LJ, Styles EC, Wilson CM. East london’s homeless: a retrospective review of an eye clinic for homeless people. BMC Health Serv Res. 2016;16(1). 10.1186/s12913-016-1295-8. PMC4754935 26880157

[79] Yu D, Cai W, Shen T, PM2.5 exposure increases dry eye disease risks through corneal epithelial inflammation and mitochondrial dysfunctions. Cell Biol Toxicol. 2023;39(6):2615–2630. 10.1007/s10565-023-09791-z. 36786954 PMC10693534

[80] Sleet DA, Francescutti LH. Homelessness and public health: a focus on strategies and solutions. IJERPH. 2021;18(21). 10.3390/ijerph182111660. PMC8583397 34770173

[81] Takahashi PY, Ryu E, Hathcock MA, A novel housing-based socioeconomic measure predicts hospitalisation and multiple chronic conditions in a community population. J Epidemiol Community Health. 2016;70(3):286–291. 10.1136/jech-2015-205925. 26458399 PMC4852846

[82] Ige MO, French DD, Chaudhury AS, Quality of care in patients with newly diagnosed glaucoma. JAMA Ophthalmol. 2025. 10.1001/jamaophthalmol.2025.2995. PMC12447283 40965904

[83] Coube M, Nikoloski Z, Mrejen M, Mossialos E. Inequalities in unmet need for health care services and medications in Brazil: a decomposition analysis. Lancet Reg Health Am. 2023;19, 100426. 10.1016/j.lana.2022.100426. 36950032 PMC10025415

[84] Johnson-Griggs MA, Hicks PM, Lu MC, Relationship between unstable housing, food insecurity, and vision status in the MI-SIGHT community eye disease screening program. Ophthalmology. 2024;131(2):140–149. 10.1016/j.ophtha.2023.09.011. 37709171 PMC11044600

