# Supplementary material for: Assessing the 3 pillars of housing for eye and vision health outcomes: A scoping review
Source: Surv Ophthalmol. Author manuscript; Available in PMC 2026 Apr 16. (PMC13084688; doi:10.1016/j.survophthal.2025.12.008)
Supplement: 1 [file NIHMS2159711-supplement-1.docx]

**Supplemental Table 1. Explored and Observed Pillars of Housing by Study**

| **Publication/Location/ Study Method** | **Eye Conditions** | **Explored*** | **Observed** |
| --- | --- | --- | --- |
| **Dry Eye** | | | |
| Kaplan et al. 2019/USA/Cross-Sectional | 1. Dry Eye | Conditions:   1. Particulate Matter exposure 2. Indoor temperature 3. Relative humidity | Conditions:   1. Particulate Matter exposure 2. Indoor temperature |
| Huang et al. 2021/USA/Cross-Sectional | 1. Dry Eye | Conditions:   1. Humidity levels 2. Particulate Matter exposure (PM concentration: PM2.5 or PM10 and PM count) 3. Indoor temperature 4. Seasonality | Conditions:   1. Humidity levels 2. Particulate Matter exposure (PM concentration: PM2.5 or PM10 and PM count) |
| Rock et al. 2022/USA/Cross-Sectional | 1. Dry Eye (Corneal epithelial disruption, Lower eyelid meibomian gland dropout) | Conditions:   1. Age of home 2. Indoor humidity 3. Indoor temperature 4. Number of microbial colonies (CFU) in the home | Conditions:   1. Number of microbial colonies (CFU) in the home |
| **Glaucoma** | | | |
| Bhorade et al. 2013/USA/Cross-Sectional | 1. Glaucoma | Conditions:   1. Lighting levels | Conditions:   1. Lighting levels |
| Ramulu et al. 2022/USA/Prospective Cohort Study | 1. Glaucoma | Conditions:   1. Lighting levels 2. Home hazards | Conditions:   1. Lighting levels |
| Yonge et al. 2018/USA/Cross-Sectional | 1. Glaucoma | Conditions:   1. Home hazards (ambient lighting <300 lux, exposed light bulbs, lack of grab bars by the toilet, door threshold height > 0.5 inches, seats without arm rests) | Conditions:   1. Not observed. |
| **Cataract** | | | |
| Vashist et al. 2020/India/Cross-Sectional | 1. Cataract (nuclear, cortical, posterior subcapsular) | Conditions:   1. Sun exposure 2. Indoor kitchen smoke exposure | Conditions:   1. Sun exposure 2. Indoor kitchen smoke exposure |
| Quintana et al. 2013/Spain/Prospective Cohort Study | 1. Cataract | Consistency:   1. Living arrangements (living alone, with a partner, or in a residential home) | Consistency:   1. Living arrangements (in a residential home) |
| **Diabetic Retinopathy** | | | |
| Thomas et al. 2021/UK/Retrospective Observational Study | 1. Diabetic Retinopathy | Consistency:   1. Frequency of house moves | Consistency:   1. Frequency of house moves |
| Chauhan et al. 2020/India/Cross-Sectional | 1. Diabetic Retinopathy | Conditions:   1. Dwelling Altitude | Conditions:   1. Dwelling Altitude |
| Silverberg et al. 2021/USA/Cross-Sectional | 1. Diabetic Retinopathy | Consistency:   1. Homeownership (Own or Rent) | Consistency:   1. Not observed. |
| Davis et al. 2017/USA/Longitudinal Cohort Study | 1. Diabetic Retinopathy | Consistency:   1. Homelessness | Consistency:   1. Homelessness |
| Cai et al. 2021/USA/Prospective Cohort Study | 1. Diabetic Retinopathy | Conditions:   1. Poor housing conditions (e.g., overcrowded, inadequate heating) | Conditions:   1. Poor housing conditions (e.g., overcrowded, inadequate heating) |
| **Retinal Vein Occlusion** | | | |
| McDermott et al. 2022/USA/Retrospective Case-Control Study | 1. Retinal Vein Occlusion (Branch and Central) | Consistency:   1. Increasing years living at current address 2. Currently rent or own home 3. Currently living with a partner | Consistency:   1. Increasing years living at current address 2. Currently rent or own home 3. Currently living with a partner |
| **Neuromyelitis Optica Spectrum Disorder** | | | |
| Rafiee et al. 2020/Iran/Case-Control Study | 1. Neuromyelitis Optica Spectrum Disorder | Consistency:   1. Homelessness periods | Consistency:   1. Homelessness periods |
| **Ocular Toxoplasmosis** | | | |
| Abu et al. 2016/Ghana/Community-based Epidemiological Study | 1. Ocular Toxoplasmosis | Conditions:   1. Dispose of cat litter 2. Cat ownership 3. Contact with soil 4. Rural dwelling | Conditions:   1. Not observed. |
| **Exfoliation Syndrome** | | | |
| Aygun et al. 2023/Turkey/Case-control Study | 1. Exfoliation Syndrome 2. Exfoliation Glaucoma | Conditions:   1. Type of Housing 2. Heating Method (Stove or central heating system) | Conditions:   1. Type of Housing 2. Heating Method (Stove) |
| **Amblyopia** | | | |
| Bountziouka et al. 2021/England/Cross-Sectional | 1. Persisting Unilateral Amblyopia | Consistency:   1. Housing tenure (rented vs owned) | Consistency:   1. Not observed. |
| **Conjunctivitis** | | | |
| Reboux et al. 2018/France/Cross-Sectional | 1. Conjunctivitis | Conditions:   1. Water damage 2. Outdoor and indoor temperature 3. Relative humidity 4. Mite detection 5. Water leakage 6. Age of house 7. High occupant-surface ratio 8. High number of inhabitants per cubic meter 9. Lack of ventilation 10. Electric heating 11. Apartments located on the ground floor 12. Dwellings with fewer rooms 13. Presence of pets 14. Presence of a washing machine and windows 15. Airing time per day 16. Indoor hygrometry 17. Floor covering 18. Indoor fungal contamination | Conditions:   1. Not observed. |
| Suryani et al. 2021/Indonesia/Case-Control | 1. Conjunctivitis | Conditions:   1. Distance between house and river (less/greater than 250 m) 2. Distance between house and temporary garbage dump (less/greater than 250 m) 3. Window always open daily | Conditions:   1. Distance between house and river (less/greater than 250 m) 2. Distance between house and temporary garbage dump (less/greater than 250 m) 3. Window always open daily |
| **Trachoma** | | | |
| Debrah et al. 2017/Ghana/Cross-Sectional | 1. Trachoma (Trachomatous inflammation-follicular in children 1-9 years, Trachomatous trichiasis in adults 15 years or older) | Conditions:   1. Sanitation facilities 2. Hygiene practices 3. Access to water | Conditions:   1. Not observed. |
| Chen et al. 2021/Tanzania/Cross-Sectional | 1. Trachoma | Conditions:   1. Cleanliness index (clean yard, improved latrine, washing clothes, at least 1 child in the household having a clean face) 2. Crowding 3. Availability of piped water | Conditions:   1. Cleanliness index (clean yard, improved latrine, washing clothes, at least 1 child in the household having a clean face) |
| Silva et al. 2020/Brazil/Cross-Sectional | 1. Trachoma | Conditions:   1. Living in unfinished houses 2. Absence of a sewage system 3. Presence of bathroom, electric shower, water, and electricity 4. Type of roof and flooring 5. Number of bedrooms   Consistency:   1. Property type (Own vs. Rented) 2. Living with relatives | Conditions:   1. Living in unfinished houses 2. Absence of a sewage system   Consistency:   1. Not observed. |
| **Eye Irritation** | | | |
| Youssef et al. 2021/Morocco/Cross-Sectional | 1. Eye irritation   Blurred vision | Conditions:   1. Age of building (in years) 2. Number of hours spent in the home 3. Occupied floor 4. Type of housing (single family, bifamily, multifamily) 5. Number of sides 6. Window size in m^2^ 7. Living near a heavy traffic road, agricultural site, polluting company and type of polluting company 8. Presence of parking with heavy road traffic 9. Presence of a yard 10. Presence of construction site inside the house 11. Presence of dust in the house 12. Texture of house 13. House orientation 14. Presence of a manual vacuum cleaner, central vacuum cleaner, and cooker hood in the house 15. Dust extraction 16. Ventilation 17. Bare flooring 18. Grass siding | Conditions:   1. Age of building (in years) 2. Number of hours spent in the home 3. Occupied floor 4. Type of housing (single family, bifamily, multifamily) 5. Number of sides 6. Window size in m^2^ 7. Living near a polluting company 8. Presence of a yard 9. Presence of construction site inside the house 10. Presence of dust in the house 11. House orientation 12. Dust extraction 13. Ventilation 14. Bare flooring 15. Grass siding |
| Zhang et al. 2018/China/Cross-Sectional | 1. Itching, burning, or irritation of the eyes | Conditions:   1. Damp stains on floor, walls, or ceiling 2. Mold spots on floor, walls, or ceiling 3. House size 4. Construction year of the building 5. Damp bed clothing in the last year 6. Water damage 7. Window pane condensation 8. Moldy odor indoors in the last 3 months 9. Perception of humid air | Conditions:   1. Damp stains on floor, walls, or ceiling 2. Mold spots on floor, walls, or ceiling 3. Perception of humid air |
| Johnston et al. 2022/USA/Cross-Sectional | 1. Eye irritation | Conditions:   1. Living near active oil development site | Conditions:   1. Living near active oil development site |
| Yang et al. 2021/Sweden/Cross-Sectional | 1. Itching, Burning, or Irritation of the eyes | Conditions:   1. Number of persons living in the dwelling 2. Floor area of dwelling 3. Type of ventilation system 4. Frequency of window opening in heating season 5. Environmental tobacco smoke at home 6. Condensation on window panes in winter 7. In the last 12 months: Water leakage, Floor dampness, Any dampness, Visible molds, Mold odor, Any odor other than mold odor, Recent indoor painting, New floor materials installed 8. Wall-to-wall carpets and oiled wooden floors in any room 9. Presence of electric radiators for heating   Consistency:   1. Ownership of dwelling (Self-owned/rented) | Conditions:   1. In the last 12 months: Floor dampness, Any dampness, Visible molds 2. Type of ventilation system 3. Number of persons living in the dwelling 4. Environmental tobacco smoke at home 5. Presence of electric radiators for heating   Consistency:   1. Not observed. |
| **Eye Infections** | | | |
| Firdaus et al. 2012/India/Cross-Sectional | 1. Eye infections | Conditions:   1. Overcrowding 2. Dampness 3. Moldy conditions 4. Indoor air pollution 5. Poor structural condition of house | Conditions:   1. Indoor air pollution 2. Poor structural condition of house |
| Lopez et al. 2024/USA/Retrospective Case-Control Study | 1. Infectious keratitis | Consistency:   1. Housing status (Housed, Homeless, Unknown) | Consistency:   1. Housing status (Housed, Homeless, Unknown) |
| Carnt et al. 2020/UK/Cross-Sectional | 1. Acanthamoeba keratitis | Conditions:   1. Acanthamoeba and free-living amoeba colonization in water fixture locations (Overflows, drains, spouts in bathroom and kitchen) | Conditions:   1. Free-living amoeba colonization in the bathroom’s overflows and kitchen’s drain |
| **Eyesight** | | | |
| Melody et al. 2016/Australia/Cross-Sectional | 1. Eyesight | Conditions:   1. Overcrowding 2. Dust | Conditions:   1. Overcrowding 2. Dust |
| Shiue et al. 2015/USA/Cross-Sectional | 1. Vision (Good, Fair, Poor) | Conditions:   1. Indoor mildew odor or musty smell 2. Age of housing | Conditions:   1. Indoor mildew odor or musty smell |
| **Visual Impairment** | | | |
| Lee et al. 2018/Korea/Cross-Sectional | 1. Vision Impairment | Conditions:   1. Slippery bathroom floors 2. Sharp edges of furniture or walls 3. Floor level differences in interior spaces 4. Obstacles and hazards in corridors and pathways 5. Difficulties and inconveniences within various areas of the home like the living room, kitchen, bedroom, bathroom, and laundry area 6. Issues with using housing appliance and locating things 7. Lighting in interior space and outside house/ Lighting Conditions 8. Proper heating and ventilation | Conditions:   1. Slippery bathroom floors 2. Sharp edges of furniture or walls 3. Floor level differences in interior spaces 4. Obstacles and hazards in corridors and pathways 5. Difficulties and inconveniences within various areas of the home like the living room, kitchen, bedroom, bathroom, and laundry area 6. Issues with using housing appliance issues and locating things 7. Lighting Conditions |
| Brown et al. 2016/USA/Cross-Sectional | 1. Visual impairment | Consistency:   1. Unsheltered 2. Lived in multiple locations including hotels and shelters 3. Staying with family and friends 4. Recently homeless | Consistency:   1. Recently homeless |
| Lebrun-Harris et al. 2012/USA/Cross-Sectional | 1. Vision impairment | Consistency:   1. Number of homeless episodes in a lifetime 2. Current living situation (ex. own place, emergency shelter, transitional shelter, hotel or motel) | Consistency:   1. Not observed. |
| Pesonen et al. 2022/Finland/Cross-Sectional | 1. Visual impairment | Conditions:   1. Home-dwelling and receiving home care | Conditions:   1. Home-dwelling and receiving home care |
| Tham et al. 2018/Singapore/Cross-Sectional | 1. Uni/Bilateral Visual impairment 2. Uni/Bilateral Blindness | Conditions:   1. Type of housing (1-2, 3-4, greater than 5 room public housing flat) | Conditions:   1. Type of housing (1-2, 3-4, greater than 5 room public housing flat) |
| Pooprasert et al. 2020/UK/Cross-Sectional | 1. Refractive error 2. Visual impairment 3. Blindness | Consistency:   1. Homelessness | Consistency:   1. Homelessness |
| Alvarado-Esquivel et al. 2015/Mexico/Cross-Sectional | 1. Visual impairment 2. Blindness | Conditions:   1. Crowding at home (number of persons and rooms in the house) 2. Type of flooring of the house (Soil floor) 3. Availability of drinkable water 4. Form of elimination of excreta | Conditions:   1. Type of flooring of the house (Soil floor) |
| Wong et al. 2020/Hong Kong/Cross-Sectional | 1. Unilateral/Bilateral Visual Impairment 2. Refractive error | Consistency:   1. Type of housing (Private or temporary) | Consistency:   1. Type of housing (Temporary) |
| Andersson et al. 2020/USA/Cross-Sectional | 1. Visual impairment   Blindness | Conditions:   1. Living in a nursing home | Conditions:   1. Living in a nursing home |
| **Refractive Error** | | | |
| D’ath et al. 2016/UK/Retrospective Case Study | 1. Myopia 2. Hyperopia | Consistency:   1. Homelessness | Consistency:   1. Homelessness |
| Marmamula et al. 2020/India/Cross-Sectional | 1. Uncorrected Refractive Error | Conditions:   1. Type of home (Private, Aided/Partially aided, Free) | Conditions:   1. Type of home (Aided/Partially aided, Free) |
| **Multiple Eye Conditions** | | | |
| Haanes et al. 2015/Norway/Randomized Controlled Trial | 1. Cataract 2. Glaucoma 3. Age-related Macular Degeneration 4. Diabetic Retinopathy 5. Uncorrected Refractive Errors | Conditions:   1. Indoor lighting conditions (ex. number and placement of lamps, type of lighting, brightness and type of light bulbs used) 2. Presence and reduction of glare | Conditions:   1. Indoor lighting conditions (ex. number and placement of lamps, type of lighting, brightness and type of light bulbs used) |
| Hom et al. 2021/USA/Cross-Sectional | 1. Cataract 2. Glaucoma 3. Age-related Macular Degeneration 4. Diabetic Retinopathy | Cost:   1. Housing payments | Cost:   1. Housing payments |
| Hennein et al. 2021/USA/Prospective Cohort Study | Vision-threatening diagnoses:   1. Cataract 2. Glaucoma or suspected glaucoma 3. Afferent pupillary defect 4. Visual field defect 5. Acute posterior vitreous detachment 6. Proliferative diabetic retinopathy 7. Nonproliferative diabetic retinopathy 8. Age-related macular degeneration 9. Macular hole 10. Suspicious choroidal lesion 11. Proptosis 12. Aphakia 13. Retinal detachment 14. Macular scar   Non-vision threatening diagnoses:   1. Refractive error 2. Presbyopia 3. Myopia 4. Astigmatism 5. Hyperopia 6. Dry eye syndrome 7. Epithelial basement membrane dystrophy 8. Epiphora 9. Strabismus 10. Choroidal nevus 11. Lattice degeneration 12. Ptosis 13. Blepharitis 14. Epitheliopathy | Consistency:   1. Duration of stay at the shelter 2. Stability of housing | Consistency:   1. Duration of stay at the shelter 2. Stability of housing |
| French et al. 2019/USA/Cross-Sectional | Non-traumatic disorders:   1. Diplopia 2. Various types of visual disturbances 3. Unspecified vision loss 4. Orbital cellulitis   Traumatic disorders:   1. Closed fracture of the orbital floor 2. Ocular laceration | Cost:   1. Expensive housing costs relative to income   Conditions:   1. Overcrowding 2. Inadequate Plumbing 3. Kitchen Facilities 4. Air pollution | Cost:   1. Expensive housing costs relative to income   Conditions:   1. Air pollution 2. Overcrowding |
| Slomovic et al. 2023/Canada/Cross-Sectional | 1. Cataract 2. Chalazion 3. Nonproliferative Diabetic Retinopathy (moderate, bilateral) 4. Pterygia (Bilateral) 5. Visual impairment 6. Refractive error | Conditions:   1. Marginally housed | Conditions:   1. Marginally housed |
| Abdu et al. 2013/Nigeria/Cross-Sectional | 1. Cataract 2. Glaucoma 3. Blindness 4. Severe visual impairment | Conditions:   1. Lived on the street 2. Living in uncompleted buildings   Consistency:   1. Rented Accommodation 2. Own House 3. Living with Relatives | Conditions:   1. Lived on the street 2. Living in uncompleted buildings   Consistency:   1. Not observed. |
| Wang et al. 2023/China/Cross-Sectional | 1. Cataract 2. Glaucoma 3. Age-related Macular Degeneration 4. Diabetic Retinopathy   Presbyopia | Conditions:   1. General visual conditions 2. Lighting quality 3. Design features like window placement and size 4. Arrangement and visibility of furniture, signage, and handrails 5. Contrast and color of interior designs | Conditions:   1. Lighting quality |
| Sutradhar et al. 2019/Bangladesh/Two-phase mixed methods study | 1. Refractive error 2. Conjunctivitis 3. Visual impairment   Cataract | Conditions:   1. Slum dwellers | Conditions:   1. Slum dwellers |
| Hennein et al. 2020/USA/Cross-Sectional | 1. Glaucoma 2. Diabetic Retinopathy 3. Diabetic Macular Edema 4. Hypertensive Retinopathy 5. Cataract 6. Dry Eye 7. Corneal Epitheliopathy 8. Malignancy of eyelid 9. Chorioretinal Scar 10. Bitemporal Hemianopsia 11. Stabismus 12. Diplopia 13. Duane’s Syndrome 14. Iris Coloboma 15. Pingueculitis 16. Aphakia | Consistency:   1. Number of years without housing 2. Duration of time at shelter | Consistency:   1. Not observed. |
| Suksohale et al. 2013/India/Exposure-Response Study | 1. Eye irritation 2. Diminution of vision 3. Cataract | Conditions:   1. Kitchen location 2. Adequacy of ventilation 3. Indoor air pollution 4. Exposure to indoor air pollutants from biomass combustion | Conditions:   1. Exposure to indoor air pollutants from biomass combustion |
| Elliott et al. 2019/USA/Cross-Sectional | 1. Cataract 2. Glaucoma 3. Age-related Macular Degeneration 4. Diabetic Retinopathy 5. Refractive Error | Conditions:   1. Living in subsidized senior housing (SSH) communities | Conditions:   1. Living in subsidized senior housing (SSH) communities |
| Park et al. 2024/Canada/Cross-Sectional | 1. Refractive error 2. Blindness 3. Cataract 4. Optic neuropathy 5. Amblyopia 6. Retinal hole/retinal hypopigmentation 7. Nasolacrimal duct 8. Thyroid-associated ophthalmopathy 9. Ocular rosacea 10. Ocular hypertension/suspected glaucoma 11. Narrow angles 12. Glaucoma 13. Iridectomy/Iridotomy 14. Retinal diseases/abnormality 15. Up-gaze palsy 16. Ehlers-Danlos syndrome 17. Relative afferent pupillary defect | Consistency:   1. Duration of homelessness | Consistency:   1. Not observed. |
| Jiang et al. 2020/Canada/Cross-Sectional | 1. Blindness 2. Cataract 3. Diabetic Retinopathy 4. Dry Eye 5. Floaters 6. Macular degeneration 7. Refractive error 8. Suspected glaucoma 9. Trauma-related injury 10. Corneal conditions 11. Retinopathy 12. Pseudophakia/aphakia 13. Vitreous problem 14. Ectropion or entropion 15. Eye movement disorder 16. Pinguecula/pterygium | Consistency:   1. Living in homeless shelters | Consistency:   1. Living in homeless shelters |
| Low et al. 2020/Singapore/Cohort Study | 1. Visual impairment 2. Diabetic Retinopathy | Conditions:   1. Poor living arrangement 2. Person-level socioeconomic status (PLSES) (ex. Housing type (small-sized public apartments: 1-2 rooms, medium-sized public apartments: up to 4 rooms, large public apartments: 5 rooms, or private housing) | Conditions:   1. Poor living arrangement 2. Person-level socioeconomic status (PLSES) (ex. Housing type (small-sized public apartments: 1-2 rooms, medium-sized public apartments: up to 4 rooms) |
| Yelle et al. 2022/Canada/Cross-Sectional | 1. Visual impairment 2. Uncorrected Refractive Error 3. Cataract 4. Age-related Macular Degeneration 5. Glaucoma 6. Diabetic Retinopathy 7. Presbyopia | Consistency:   1. Homelessness | Consistency:   1. Homelessness |
| Sawers et al. 2016/UK/Cross-Sectional | 1. Visual impairment 2. Blindness 3. Cataract and other lens problems 4. Vitreoretinal problems 5. External eye disease 6. Ocular motility problems 7. Medical retina 8. Glaucoma 9. Orbit and eyelid problems 10. Refractive error 11. Optic nerve disease | Consistency:   1. Homelessness | Consistency:   1. Homelessness |
| Marmamula et al. 2023/India/Cross-Sectional | 1. Unilateral Vision Loss caused by cataract 2. Uncorrected Refractive Error 3. Posterior capsular opacification 4. Posterior segment disease 5. Glaucoma 6. Corneal disease | Conditions:   1. Type of home (Private, Aided/Partially aided, Free) | Conditions:   1. Type of home (Free) |
| Yekta et al. 2019/Iran/Cross-Sectional | 1. Visual impairment 2. Blindness 3. Refractive errors 4. Cataract 5. Glaucoma 6. One eyed caused by injury 7. Corneal opacity | Conditions:   1. Living in a nursing home | Conditions:   1. Living in a nursing home |
| Noel et al. 2015/Canada/Cross-Sectional | 1. Visual impairment due to suspected glaucoma 2. Cataract 3. Blocked nasolacrimal duct 4. Dry age-related macular degeneration 5. Fourth nerve palsy 6. Homonymous hemianopsia secondary to stroke 7. Nonproliferative Diabetic Retinopathy 8. Penetrating keratoplasty graft rejection 9. Traumatic cataract 10. Idiopathic corneal opacity | Consistency:   1. Homelessness | Consistency:   1. Homelessness |
| Ryu et al. 2018/USA/Longitudinal Cohort Study | 1. Eye diseases | Conditions:   1. HOUsing-based SocioEconomic Status (HOUSES) Index | Conditions:   1. HOUsing-based SocioEconomic Status (HOUSES) Index |

***** “Explored” refers to variables measured in the included studies. “Observed” refers to variables that showed a reported association with ocular outcomes. PM2.5 and PM10 refer to particulate matter <2.5 μm and <10 μm in diameter, respectively.
