# Supplementary material for: Assessing the 3 pillars of housing for eye and vision health outcomes: A scoping review
Source: Surv Ophthalmol. Author manuscript; Available in PMC 2026 Apr 16. (PMC13084688; doi:10.1016/j.survophthal.2025.12.008)
Supplement: 2 [file NIHMS2159711-supplement-2.docx]

**Supplemental Table 2. Codebook for Explored and Observed Risk Factors by Pillars of Housing**

| **Pillars of Housing** | **Explored Risk Factors*** | **Observed Risk Factors** |
| --- | --- | --- |
| **Cost** | - Housing payments - Expensive housing costs relative to income | - Housing payments - Expensive housing costs relative to income |
| **Conditions** | - Outdoor and Indoor temperature - Relative humidity - Seasonality - Age of home - Number of microbial colonies (CFU) in the home - Dwelling Altitude - Occupied floor - Sanitation facilities (plumbing, form of elimination of excreta) - Hygiene practices - Cleanliness index (clean yard, improved latrine, washing clothes, at least 1 child in the household having a clean face) - Presence of bathroom, electric shower, water, electricity, yard, construction site inside the house, wall-to-wall carpets and oiled wooden floors in any room, electric radiators or stove for heating, central heating system, washing machine, drinkable water) - Number of hours spent in the home - Dust extraction - Grass siding - Acanthamoeba and free-living amoeba colonization in water fixture locations (Overflows, drains, spouts in bathroom and kitchen) - Issues with using housing appliances and locating items - Lighting conditions (indoor and outdoor, number and placement of lamps, type of lighting, brightness and type of light bulbs used, general visual conditions, presence and reduction of glare) - Person-level socioeconomic status (PLSES) - Type of housing (single family, bifamily, multifamily, 1-2, 3-4, greater than 5 room public housing flat, private, aided/partially aided, free) - Living arrangements (nursing home, subsidized senior housing communities, on the street, uncompleted buildings, marginally housed, home-dwelling and receiving home care, slum dwellers, number of persons living in the dwelling) - Housing structure (house size, type of roof and flooring: soil/bare, house orientation, texture of house, number of sides, window size in m^2^, window placement, arrangement and visibility of furniture, signage, and handrails, contrast and color of interior designs, kitchen location and facilities, poor structural condition) - Housing location (proximity to heavy traffic roads, agricultural sites, polluting companies, active oil development site, rivers, temporary garbage dumps) - Environmental exposure (Particulate Matter: PM concentration- PM2.5 or PM10 and PM count, sun exposure, indoor kitchen smoke, indoor air pollution from biomass combustion, indoor fungal contamination, dust, mites, damp stains, contact with soil, mold in the last 12 months, moldy odor in the last 12 months, any odor other than moldy odor, cat litter, tobacco smoke) - Home hazards (ambient lighting <300 lux, exposed light bulbs, lack of grab bars by the toilet, door threshold height > 0.5 inches, seats without arm rests, slippery bathroom floors, sharp edges of furniture or walls, floor level differences in interior spaces, obstacles in pathways) - Issues with using housing appliances and locating items - Living space conditions (overcrowding, number of bedrooms, floor area, high occupant-surface ratio, high number of inhabitants per cubic meter) - Ventilation and air quality (type of ventilation system, airing time per day, indoor hygrometry, presence of a manual or central vacuum cleaner, cooker hood, window always open daily, frequency of window opening in heating season) - Home maintenance (window pane condensation, in the last 12 months: water leakage, any dampness, recent indoor painting, new floor materials installed, presence of damp bed clothing) - HOUsing-based SocioEconomic Status (HOUSES) Index | - Indoor temperature - Humidity levels - Number of microbial colonies (CFU) in the home - Dwelling Altitude - Cleanliness index (clean yard, improved latrine, washing clothes, at least 1 child in the household having a clean face) - Presence of yard, construction site inside the house, electric radiators or stove for heating) - Absence of a sewage system - Age of building (in years) - Number of hours spent in the home - Occupied floor - Type of housing (single family, bifamily, multifamily, 1-2, 3-4, greater than 5 room public housing flat, aided/partially aided, free) - Living arrangements (nursing home, subsidized senior housing communities, on the street, uncompleted buildings, marginally housed, home-dwelling and receiving home care, slum dwellers, number of persons living in the dwelling) - Housing structure (house orientation, type of flooring: soil/bare, number of sides, window size in m^2^, poor structural condition) - Housing location (proximity to polluting company, active oil development site, rivers, temporary garbage dumps) - Environmental exposure (Particulate Matter: PM concentration- PM2.5 or PM10 and PM count, sun exposure, indoor kitchen smoke, indoor air pollution from biomass combustion, dust, damp stains, mold in the last 12 months, any odor other than moldy odor, tobacco smoke) - Home hazards (slippery bathroom floors, sharp edges of furniture or walls, floor level differences in interior spaces, obstacles in pathways) - Overcrowding - Ventilation and air quality (type of ventilation system, window always open daily) - Dampness in the last 12 months - Dust extraction - Grass siding - Free-living amoeba colonization in the bathroom’s overflows and kitchen’s drain - Issues with using housing appliances and locating items - Lighting conditions (indoor, number and placement of lamps, type of lighting, brightness and type of light bulbs used) - Person-level socioeconomic status (PLSES) (ex. Housing type (small-sized public apartments: 1-2 rooms, medium-sized public apartments: up to 4 rooms, large public apartments: 5 rooms, or private housing) - HOUsing-based SocioEconomic Status (HOUSES) Index |
| **Consistency** | - Frequency of house moves - Homeownership (Own or Rent) - Increasing years living at current address - Current living situation (private or temporary housing, emergency shelter, transitional shelter, hotel, motel, homeless, unknown) - Lived in multiple locations - Number of homeless episodes in a lifetime - Number of years without housing - Duration of stay at the shelter - Stability of housing - Living arrangements (living alone, with a partner, in a residential home, with relatives) | - Frequency of house moves - Increasing years living at current address - Homeownership (Own or Rent) - Housing status (temporary housing, homeless, unknown) - Duration of stay at the shelter - Stability of housing - Living arrangements (in a residential home) |

***** “Explored” refers to variables measured in the included studies. “Observed” refers to variables that showed a reported association with ocular outcomes. PM2.5 and PM10 refer to particulate matter <2.5 μm and <10 μm in diameter, respectively.
