# Supplementary material for: Assessing the 3 pillars of housing for eye and vision health outcomes: A scoping review
Source: Surv Ophthalmol. Author manuscript; Available in PMC 2026 Apr 16. (PMC13084688; doi:10.1016/j.survophthal.2025.12.008)
Supplement: 3 [file NIHMS2159711-supplement-3.docx]

**Supplemental Table 3. Explored vs. Observed Pillars of Housing in all Studies**

| **Explored: Conditions** | **Percent Total Observed*** |
| --- | --- |
| Pollution (8) | 100% |
| Humidity (5) | 60% |
| Temperature (4) | 25% |
| Home Hazards (9) | 44.4% |
| Home Age (5) | 20% |
| Lighting (5) | 100% |
| Location (8) | 50% |
| Home Type (10) | 100% |
| Number of persons in the home (10) | 40% |
| Home Cleanliness (9) | 56% |
| Home Damage (2) | 0% |
| Proper heating and ventilation (8) | 37.5% |
| Marginally housed (3) | 100% |
| **Explored: Consistency** | **Percent Total Observed** |
| Number of persons in the home (5) | 40% |
| Home Ownership (8) | 25% |
| Location of home (1) | 100% |
| Length of stay in home (4) | 50% |
| Housing Status (13) | 77% |
| **Explored: Cost** | **Percent Total Observed** |
| Housing Payments (2) | 100% |

***** “Percent Total Observed” refers to the proportion of studies exploring a given factor in which a statistically significant association with ocular outcomes was reported.
