# Supplementary material for: Assessing the 3 pillars of housing for eye and vision health outcomes: A scoping review
Source: Surv Ophthalmol. Author manuscript; Available in PMC 2026 Apr 16. (PMC13084688; doi:10.1016/j.survophthal.2025.12.008)
Supplement: 4 [file NIHMS2159711-supplement-4.docx]

**Supplemental Figure 1.** Scoping Review Databases and Search Strategies

| **PubMed**  1. "Eye"[Mesh] OR "Ophthalmology"[Mesh] OR "Optometry"[Mesh] OR "Vision Disorders"[Mesh] OR "Eye Diseases"[Mesh] OR "Visually Impaired Persons"[Mesh] OR "Vision, Ocular"[Mesh] OR "Diagnostic Techniques, Ophthalmological"[Mesh] OR "Ophthalmologic Surgical Procedures"[Mesh] OR "Ophthalmologists"[Mesh] OR "Optometrists"[Mesh] OR "aicardi syndrome"[tiab] OR "amaurosis fugax"[tiab] OR "angioid streaks"[tiab] OR "artificial lens"[tiab] OR "cogan syndrome"[tiab] OR "color perception"[tiab] OR "cone dystrophy"[tiab] OR "duane retraction syndrome"[tiab] OR "ectopia lentis"[tiab] OR "epiretinal membrane"[tiab] OR "familial exudative vitreoretinopathies"[tiab] OR "fluorescein angiography"[tiab] OR "leber congenital amaurosis"[tiab] OR "lens subluxation"[tiab] OR "limbal stem cell deficiency"[tiab] OR "meibomian gland"[tiab] OR "miller fisher syndrome"[tiab] OR "Mucous Membrane Pemphigoid" [tiab] OR "opsoclonus-myoclonus"[tiab] OR "orbital cellulitis"[tiab] OR "orbital disease*"[tiab] OR "orbital myositis"[tiab] OR "orbital neoplasm*"[tiab] OR "orbital pseudotumor"[tiab] OR "persistent hyperplastic primary vitreous"[tiab] OR "refractive error*"[tiab] OR "retrobulbar hemorrhage"[tiab] OR "slit lamp microscopy"[tiab] OR "stargardt disease"[tiab] OR "susac syndrome"[tiab] OR "tolosa-hunt syndrome"[tiab] OR "vitreous detachment"[tiab] OR "vitreous hemorrhage"[tiab] OR "walker-warburg syndrome"[tiab] OR "weill-marchesani syndrome"[tiab] OR aberrometry[tiab] OR amblyopia[tiab] OR aniridia[tiab] OR aniseikonia[tiab] OR anisocoria[tiab] OR anisometropia[tiab] OR anophthalmos[tiab] OR aphakia[tiab] OR asthenopia[tiab] OR astigmatism[tiab] OR blephar*[tiab] OR blind*[tiab] OR cataract*[tiab] OR chalazion[tiab] OR chorioretinopath*[tiab] OR choroid*[tiab] OR coloboma[tiab] OR cone-rod[tiab] OR conjunctiv*[tiab] OR cornea*[tiab] OR dacryocystitis[tiab] OR diplopia[tiab] OR ectropion[tiab] OR electronystagmography[tiab] OR electrooculography[tiab] OR electroretinography[tiab] OR endophthalmitis[tiab] OR enophthalmos[tiab] OR entropion[tiab] OR exophthalmos[tiab] OR fluorophotometry[tiab] OR glaucoma[tiab] OR gonioscopy[tiab] OR hemianopsia[tiab] OR hordeolum[tiab] OR hydrophthalmos[tiab] OR hyperopia[tiab] OR hyphema[tiab] OR iridocorneal[tiab] OR iris[tiab] OR keratiti*[tiab] OR keratoconus[tiab] OR lacrimal[tiab] OR macula*[tiab] OR microphthalmos[tiab] OR miosis[tiab] OR mydriasis[tiab] OR myopia[tiab] OR nystagmus[tiab] OR ocular*[tiab] OR ophthalmodynamometry[tiab] OR ophthalmolog* [tiab] OR ophthalmopathy[tiab] OR ophthalmoplegia[tiab] OR ophthalmoscopy[tiab] OR optic[tiab] OR optometry [tiab] OR papilledema[tiab] OR photophobia[tiab] OR pinguecula[tiab] OR presbyopia[tiab] OR pterygium[tiab] OR pupil[tiab] OR retina*[tiab] OR retinitis[tiab] OR retinoblastoma[tiab] OR retinopath*[tiab] OR retinoscopy[tiab] OR sclera*[tiab] OR scleritis[tiab] OR scotoma[tiab] OR strabismus[tiab] OR trachoma[tiab] OR trichiasis[tiab] OR uveal*[tiab] OR uveitis[tiab] OR visual[tiab] OR vitreoretinopathy[tiab] OR xerophthalmia[tiab] OR Eye[title] OR eyes[title] OR vision[title]  2. "Housing Instability"[Mesh] OR "Ill-Housed Persons"[Mesh] OR "Residential Facilities"[Mesh] OR "Housing"[Mesh:NoExp] OR "Almshouses"[Mesh] OR "Housing for the Elderly"[Mesh] OR "Public Housing"[Mesh] OR "accessory dwelling unit"[tiab] OR "displaced people"[tiab] OR "displaced person*"[tiab] OR "home moves"[tiab] OR "residential instabil*"[tiab] OR "street people"[tiab] OR apartment*[tiab] OR condo[tiab] OR condominium*[tiab] OR condos[tiab] OR dweller*[tiab] OR dwelling[tiab] OR evicted[tiab] OR eviction*[tiab] OR foreclos*[tiab] OR homeless*[tiab] OR homeownership[tiab] OR homes[tiab] OR house[tiab] OR houses[tiab] OR housing[tiab] OR leaseholder*[tiab] OR rent[tiab] OR rental[tiab] OR renter*[tiab] OR renting[tiab] OR tenant[tiab] OR tenants[tiab] OR townhouse*[tiab] OR unhoused[tiab]  3. (animals[mesh] NOT humans[mesh])  (1 AND 2) NOT 3 |
| --- |
| **Embase** (Elsevier)  1. 'ophthalmology'/exp OR 'optometry'/exp OR 'visual disorder'/exp OR 'eye'/exp OR 'eye disease'/exp OR 'visually impaired person'/exp OR 'vision'/exp OR 'visual system examination'/exp OR 'eye surgery'/exp OR 'ophthalmologist'/exp OR 'optometrist'/exp OR ("aicardi syndrome" OR "amaurosis fugax" OR "angioid streaks" OR "artificial lens" OR "cogan syndrome" OR "color perception" OR "cone dystrophy" OR "duane retraction syndrome" OR "ectopia lentis" OR "epiretinal membrane" OR "familial exudative vitreoretinopathies" OR "fluorescein angiography" OR "leber congenital amaurosis" OR "lens subluxation" OR "limbal stem cell deficiency" OR "meibomian gland" OR "miller fisher syndrome" OR "Mucous Membrane Pemphigoid"  OR "opsoclonus-myoclonus" OR "orbital cellulitis" OR "orbital disease*" OR "orbital myositis" OR "orbital neoplasm*" OR "orbital pseudotumor" OR "persistent hyperplastic primary vitreous" OR "refractive error*" OR "retrobulbar hemorrhage" OR "slit lamp microscopy" OR "stargardt disease" OR "susac syndrome" OR "tolosa-hunt syndrome" OR "vitreous detachment" OR "vitreous hemorrhage" OR "walker-warburg syndrome" OR "weill-marchesani syndrome" OR aberrometry OR amblyopia OR aniridia OR aniseikonia OR anisocoria OR anisometropia OR anophthalmos OR aphakia OR asthenopia OR astigmatism OR blephar* OR blindness OR cataract* OR chalazion OR chorioretinopath* OR choroid* OR coloboma OR cone-rod OR conjunctiv* OR cornea* OR dacryocystitis OR diplopia OR ectropion OR electronystagmography OR electrooculography OR electroretinography OR endophthalmitis OR enophthalmos OR entropion OR exophthalmos OR fluorophotometry OR glaucoma OR gonioscopy OR hemianopsia OR hordeolum OR hydrophthalmos OR hyperopia OR hyphema OR iridocorneal OR iris OR keratiti* OR keratoconus OR lacrimal OR macula* OR microphthalmos OR miosis OR mydriasis OR myopia OR nystagmus OR ocular* OR ophthalmodynamometry OR ophthalmolog* OR  ophthalmopathy OR ophthalmoplegia OR ophthalmoscopy OR optic OR optometry OR  papilledema OR photophobia OR pinguecula OR presbyopia OR pterygium OR pupil OR retina* OR retinitis OR retinoblastoma OR retinopath* OR retinoscopy OR sclera* OR scleritis OR scotoma OR strabismus OR trachoma OR trichiasis OR uveal* OR uveitis OR visual OR vitreoretinopathy OR xerophthalmia):ti,ab OR (eye OR eyes OR vision):ti  2. 'residential home'/exp OR 'housing'/exp OR 'home for the aged'/exp OR 'housing instability'/exp OR 'homeless person'/exp OR 'eviction'/exp OR ("accessory dwelling unit" OR "displaced people" OR "displaced person*" OR "home moves" OR "residential instabil*" OR "street people" OR apartment* OR condo OR condominium* OR condos OR dweller* OR dwelling OR evicted OR eviction* OR foreclos* OR homeless* OR homeownership OR homes OR house OR houses OR housing OR leaseholder* OR rent OR rental OR renter* OR renting OR tenant OR tenants OR townhouse* OR unhoused):ti,ab  3. (('animal'/exp or 'nonhuman'/exp) NOT 'human'/exp)  (1 AND 2) NOT 3 |
| **CINAHL Complete (EBSCO)**  1. (MH "Ophthalmology") OR (MH "Diagnosis, Eye+") OR (MH "Eye Manifestations+") OR (MH "Eye Surgery+") OR (MH "Eye+") OR (MH "Eye Infections, Viral+") OR (MH "Eye Diseases+") OR (MH "Blindness") OR (MH "Vision Disorders+") OR (MH "Vision+") OR (MH "Vision Tests+") OR (MH "Vision Screening") OR (MH "Insurance, Vision") OR (MH "Optometry") OR (MH "Ophthalmologists") OR (MH "Ophthalmic Technologists") OR (MH "Optometrists") OR TI("aicardi syndrome" OR "amaurosis fugax" OR "angioid streaks" OR "artificial lens" OR "cogan syndrome" OR "color perception" OR "cone dystrophy" OR "duane retraction syndrome" OR "ectopia lentis" OR "epiretinal membrane" OR "familial exudative vitreoretinopathies" OR "fluorescein angiography" OR "leber congenital amaurosis" OR "lens subluxation" OR "limbal stem cell deficiency" OR "meibomian gland" OR "miller fisher syndrome" OR "Mucous Membrane Pemphigoid"  OR "opsoclonus-myoclonus" OR "orbital cellulitis" OR "orbital disease*" OR "orbital myositis" OR "orbital neoplasm*" OR "orbital pseudotumor" OR "persistent hyperplastic primary vitreous" OR "refractive error*" OR "retrobulbar hemorrhage" OR "slit lamp microscopy" OR "stargardt disease" OR "susac syndrome" OR "tolosa-hunt syndrome" OR "vitreous detachment" OR "vitreous hemorrhage" OR "walker-warburg syndrome" OR "weill-marchesani syndrome" OR aberrometry OR amblyopia OR aniridia OR aniseikonia OR anisocoria OR anisometropia OR anophthalmos OR aphakia OR asthenopia OR astigmatism OR blephar* OR blind* OR cataract* OR chalazion OR chorioretinopath* OR choroid* OR coloboma OR cone-rod OR conjunctiv* OR cornea* OR dacryocystitis OR diplopia OR ectropion OR electronystagmography OR electrooculography OR electroretinography OR endophthalmitis OR enophthalmos OR entropion OR exophthalmos OR fluorophotometry OR glaucoma OR gonioscopy OR hemianopsia OR hordeolum OR hydrophthalmos OR hyperopia OR hyphema OR iridocorneal OR iris OR keratiti* OR keratoconus OR lacrimal OR macula* OR microphthalmos OR miosis OR mydriasis OR myopia OR nystagmus OR ocular* OR ophthalmodynamometry OR ophthalmolog* OR  ophthalmopathy OR ophthalmoplegia OR ophthalmoscopy OR optic OR optometry OR  papilledema OR photophobia OR pinguecula OR presbyopia OR pterygium OR pupil OR retina* OR retinitis OR retinoblastoma OR retinopath* OR retinoscopy OR sclera* OR scleritis OR scotoma OR strabismus OR trachoma OR trichiasis OR uveal* OR uveitis OR visual OR vitreoretinopathy OR xerophthalmia OR eye OR eyes OR vision) OR AB("aicardi syndrome" OR "amaurosis fugax" OR "angioid streaks" OR "artificial lens" OR "cogan syndrome" OR "color perception" OR "cone dystrophy" OR "duane retraction syndrome" OR "ectopia lentis" OR "epiretinal membrane" OR "familial exudative vitreoretinopathies" OR "fluorescein angiography" OR "leber congenital amaurosis" OR "lens subluxation" OR "limbal stem cell deficiency" OR "meibomian gland" OR "miller fisher syndrome" OR "Mucous Membrane Pemphigoid"  OR "opsoclonus-myoclonus" OR "orbital cellulitis" OR "orbital disease*" OR "orbital myositis" OR "orbital neoplasm*" OR "orbital pseudotumor" OR "persistent hyperplastic primary vitreous" OR "refractive error*" OR "retrobulbar hemorrhage" OR "slit lamp microscopy" OR "stargardt disease" OR "susac syndrome" OR "tolosa-hunt syndrome" OR "vitreous detachment" OR "vitreous hemorrhage" OR "walker-warburg syndrome" OR "weill-marchesani syndrome" OR aberrometry OR amblyopia OR aniridia OR aniseikonia OR anisocoria OR anisometropia OR anophthalmos OR aphakia OR asthenopia OR astigmatism OR blephar* OR blind* OR cataract* OR chalazion OR chorioretinopath* OR choroid* OR coloboma OR cone-rod OR conjunctiv* OR cornea* OR dacryocystitis OR diplopia OR ectropion OR electronystagmography OR electrooculography OR electroretinography OR endophthalmitis OR enophthalmos OR entropion OR exophthalmos OR fluorophotometry OR glaucoma OR gonioscopy OR hemianopsia OR hordeolum OR hydrophthalmos OR hyperopia OR hyphema OR iridocorneal OR iris OR keratiti* OR keratoconus OR lacrimal OR macula* OR microphthalmos OR miosis OR mydriasis OR myopia OR nystagmus OR ocular* OR ophthalmodynamometry OR ophthalmolog* OR  ophthalmopathy OR ophthalmoplegia OR ophthalmoscopy OR optic OR optometry OR  papilledema OR photophobia OR pinguecula OR presbyopia OR pterygium OR pupil OR retina* OR retinitis OR retinoblastoma OR retinopath* OR retinoscopy OR sclera* OR scleritis OR scotoma OR strabismus OR trachoma OR trichiasis OR uveal* OR uveitis OR visual OR vitreoretinopathy OR xerophthalmia)  2. (MH "Housing+") OR (MH "Homelessness") OR (MH "Housing Instability") OR (MH "Homeless Persons") OR TI("accessory dwelling unit" OR "displaced people" OR "displaced person*" OR "home moves" OR "residential instabil*" OR "street people" OR apartment* OR condo OR condominium* OR condos OR dweller* OR dwelling OR evicted OR eviction* OR foreclos* OR homeless* OR homeownership OR "homes" OR house OR houses OR housing OR leaseholder* OR rent OR rental OR renter* OR renting OR tenant OR tenants OR townhouse* OR unhoused) OR AB("accessory dwelling unit" OR "displaced people" OR "displaced person*" OR "home moves" OR "residential instabil*" OR "street people" OR apartment* OR condo OR condominium* OR condos OR dweller* OR dwelling OR evicted OR eviction* OR foreclos* OR homeless* OR homeownership OR "homes" OR house OR houses OR housing OR leaseholder* OR rent OR rental OR renter* OR renting OR tenant OR tenants OR townhouse* OR unhoused)  3. ((MH "Animals") NOT (MH "Human"))  (1 AND 2) NOT 3 |
| **PsycInfo (EBSCO)**  1. DE "Amblyopia" OR  DE "Autokinetic Illusion" OR  DE "Balint's Syndrome" OR  DE "Binocular Vision" OR  DE "Blindness" OR  DE "Blindsight" OR  DE "Brightness Perception" OR  DE "Cataracts" OR  DE "Color Perception" OR  DE "Color Vision Deficiency" OR  DE "Cornea" OR  DE "Dark Adaptation" OR  DE "Eye (Anatomy)" OR  DE "Eye Disorders" OR  DE "Eye Fixation" OR  DE "Face Perception" OR  DE "Fovea" OR  DE "Foveal Vision" OR  DE "Glaucoma" OR  DE "Hemianopia" OR  DE "Interocular Transfer" OR  DE "Iris (Eye)" OR  DE "Lens (Eye)" OR  DE "Linear Perspective" OR  DE "Low Vision" OR  DE "Monocular Vision" OR  DE "Nystagmus" OR  DE "Ophthalmology" OR  DE "Optic Neuritis" OR  DE "Optometrists" OR  DE "Optometry" OR  DE "Peripheral Vision" OR  DE "Pupil (Eye)" OR  DE "Refraction Errors" OR  DE "Retina" OR  DE "Retinal Eccentricity" OR  DE "Retinal Image" OR  DE "Stereoscopic Vision" OR  DE "Strabismus" OR  DE "Texture Perception" OR  DE "Tunnel Vision" OR  DE "Vision Disorders" OR  DE "Vision" OR  DE "Visual Acuity" OR  DE "Visual Contrast" OR  DE "Visual Discrimination" OR  DE "Visual Field" OR  DE "Visual Perception" OR  DE "Visual Search" OR  DE "Visual Thresholds" OR  DE "Visuospatial Ability" OR TI("aicardi syndrome" OR "amaurosis fugax" OR "angioid streaks" OR "artificial lens" OR "cogan syndrome" OR "color perception" OR "cone dystrophy" OR "duane retraction syndrome" OR "ectopia lentis" OR "epiretinal membrane" OR "familial exudative vitreoretinopathies" OR "fluorescein angiography" OR "leber congenital amaurosis" OR "lens subluxation" OR "limbal stem cell deficiency" OR "meibomian gland" OR "miller fisher syndrome" OR "Mucous Membrane Pemphigoid"  OR "opsoclonus-myoclonus" OR "orbital cellulitis" OR "orbital disease*" OR "orbital myositis" OR "orbital neoplasm*" OR "orbital pseudotumor" OR "persistent hyperplastic primary vitreous" OR "refractive error*" OR "retrobulbar hemorrhage" OR "slit lamp microscopy" OR "stargardt disease" OR "susac syndrome" OR "tolosa-hunt syndrome" OR "vitreous detachment" OR "vitreous hemorrhage" OR "walker-warburg syndrome" OR "weill-marchesani syndrome" OR aberrometry OR amblyopia OR aniridia OR aniseikonia OR anisocoria OR anisometropia OR anophthalmos OR aphakia OR asthenopia OR astigmatism OR blephar* OR blind* OR cataract* OR chalazion OR chorioretinopath* OR choroid* OR coloboma OR cone-rod OR conjunctiv* OR cornea* OR dacryocystitis OR diplopia OR ectropion OR electronystagmography OR electrooculography OR electroretinography OR endophthalmitis OR enophthalmos OR entropion OR exophthalmos OR fluorophotometry OR glaucoma OR gonioscopy OR hemianopsia OR hordeolum OR hydrophthalmos OR hyperopia OR hyphema OR iridocorneal OR iris OR keratiti* OR keratoconus OR lacrimal OR macula* OR microphthalmos OR miosis OR mydriasis OR myopia OR nystagmus OR ocular* OR ophthalmodynamometry OR ophthalmolog* OR  ophthalmopathy OR ophthalmoplegia OR ophthalmoscopy OR optic OR optometry OR  papilledema OR photophobia OR pinguecula OR presbyopia OR pterygium OR pupil OR retina* OR retinitis OR retinoblastoma OR retinopath* OR retinoscopy OR sclera* OR scleritis OR scotoma OR strabismus OR trachoma OR trichiasis OR uveal* OR uveitis OR visual OR vitreoretinopathy OR xerophthalmia OR eye OR eyes OR vision) OR AB("aicardi syndrome" OR "amaurosis fugax" OR "angioid streaks" OR "artificial lens" OR "cogan syndrome" OR "color perception" OR "cone dystrophy" OR "duane retraction syndrome" OR "ectopia lentis" OR "epiretinal membrane" OR "familial exudative vitreoretinopathies" OR "fluorescein angiography" OR "leber congenital amaurosis" OR "lens subluxation" OR "limbal stem cell deficiency" OR "meibomian gland" OR "miller fisher syndrome" OR "Mucous Membrane Pemphigoid"  OR "opsoclonus-myoclonus" OR "orbital cellulitis" OR "orbital disease*" OR "orbital myositis" OR "orbital neoplasm*" OR "orbital pseudotumor" OR "persistent hyperplastic primary vitreous" OR "refractive error*" OR "retrobulbar hemorrhage" OR "slit lamp microscopy" OR "stargardt disease" OR "susac syndrome" OR "tolosa-hunt syndrome" OR "vitreous detachment" OR "vitreous hemorrhage" OR "walker-warburg syndrome" OR "weill-marchesani syndrome" OR aberrometry OR amblyopia OR aniridia OR aniseikonia OR anisocoria OR anisometropia OR anophthalmos OR aphakia OR asthenopia OR astigmatism OR blephar* OR blind* OR cataract* OR chalazion OR chorioretinopath* OR choroid* OR coloboma OR cone-rod OR conjunctiv* OR cornea* OR dacryocystitis OR diplopia OR ectropion OR electronystagmography OR electrooculography OR electroretinography OR endophthalmitis OR enophthalmos OR entropion OR exophthalmos OR fluorophotometry OR glaucoma OR gonioscopy OR hemianopsia OR hordeolum OR hydrophthalmos OR hyperopia OR hyphema OR iridocorneal OR iris OR keratiti* OR keratoconus OR lacrimal OR macula* OR microphthalmos OR miosis OR mydriasis OR myopia OR nystagmus OR ocular* OR ophthalmodynamometry OR ophthalmolog* OR  ophthalmopathy OR ophthalmoplegia OR ophthalmoscopy OR optic OR optometry OR  papilledema OR photophobia OR pinguecula OR presbyopia OR pterygium OR pupil OR retina* OR retinitis OR retinoblastoma OR retinopath* OR retinoscopy OR sclera* OR scleritis OR scotoma OR strabismus OR trachoma OR trichiasis OR uveal* OR uveitis OR visual OR vitreoretinopathy OR xerophthalmia)  2. DE "Housing" OR DE "Assisted Living" OR DE "Dormitories" OR DE "Group Homes" OR DE "Retirement Communities" OR DE "Shelters" OR DE "Homeless" OR DE "Homeless Mentally Ill" OR DE "Homeless Youth" OR DE "Human Displacement" OR TI("accessory dwelling unit" OR "displaced people" OR "displaced person*" OR "home moves" OR "residential instabil*" OR "street people" OR apartment* OR condo OR condominium* OR condos OR dweller* OR dwelling OR evicted OR eviction* OR foreclos* OR homeless* OR homeownership OR "homes" OR house OR houses OR housing OR leaseholder* OR rent OR rental OR renter* OR renting OR tenant OR tenants OR townhouse* OR unhoused) OR AB("accessory dwelling unit" OR "displaced people" OR "displaced person*" OR "home moves" OR "residential instabil*" OR "street people" OR apartment* OR condo OR condominium* OR condos OR dweller* OR dwelling OR evicted OR eviction* OR foreclos* OR homeless* OR homeownership OR "homes" OR house OR houses OR housing OR leaseholder* OR rent OR rental OR renter* OR renting OR tenant OR tenants OR townhouse* OR unhoused)  3. ((MH "Animals") NOT (MH "Human"))  ((ZP "animal") NOT (ZP "human"))  (1 AND 2) NOT 3 |
| **SocINDEX with Full Text** (EBSCO)  1. DE "OLDER people with visual disabilities" OR DE "VISION disorders -- Social aspects" OR DE "BLIND people" OR DE "PEOPLE with visual disabilities" OR DE "BLIND parents" OR DE "OLDER blind people" OR DE "BLIND women" OR DE "BLINDNESS" OR TI("aicardi syndrome" OR "amaurosis fugax" OR "angioid streaks" OR "artificial lens" OR "cogan syndrome" OR "color perception" OR "cone dystrophy" OR "duane retraction syndrome" OR "ectopia lentis" OR "epiretinal membrane" OR "familial exudative vitreoretinopathies" OR "fluorescein angiography" OR "leber congenital amaurosis" OR "lens subluxation" OR "limbal stem cell deficiency" OR "meibomian gland" OR "miller fisher syndrome" OR "Mucous Membrane Pemphigoid"  OR "opsoclonus-myoclonus" OR "orbital cellulitis" OR "orbital disease*" OR "orbital myositis" OR "orbital neoplasm*" OR "orbital pseudotumor" OR "persistent hyperplastic primary vitreous" OR "refractive error*" OR "retrobulbar hemorrhage" OR "slit lamp microscopy" OR "stargardt disease" OR "susac syndrome" OR "tolosa-hunt syndrome" OR "vitreous detachment" OR "vitreous hemorrhage" OR "walker-warburg syndrome" OR "weill-marchesani syndrome" OR aberrometry OR amblyopia OR aniridia OR aniseikonia OR anisocoria OR anisometropia OR anophthalmos OR aphakia OR asthenopia OR astigmatism OR blephar* OR blind* OR cataract* OR chalazion OR chorioretinopath* OR choroid* OR coloboma OR cone-rod OR conjunctiv* OR cornea* OR dacryocystitis OR diplopia OR ectropion OR electronystagmography OR electrooculography OR electroretinography OR endophthalmitis OR enophthalmos OR entropion OR exophthalmos OR fluorophotometry OR glaucoma OR gonioscopy OR hemianopsia OR hordeolum OR hydrophthalmos OR hyperopia OR hyphema OR iridocorneal OR iris OR keratiti* OR keratoconus OR lacrimal OR macula* OR microphthalmos OR miosis OR mydriasis OR myopia OR nystagmus OR ocular* OR ophthalmodynamometry OR ophthalmolog* OR  ophthalmopathy OR ophthalmoplegia OR ophthalmoscopy OR optic OR optometry OR  papilledema OR photophobia OR pinguecula OR presbyopia OR pterygium OR pupil OR retina* OR retinitis OR retinoblastoma OR retinopath* OR retinoscopy OR sclera* OR scleritis OR scotoma OR strabismus OR trachoma OR trichiasis OR uveal* OR uveitis OR visual OR vitreoretinopathy OR xerophthalmia OR eye OR eyes OR vision) OR AB("aicardi syndrome" OR "amaurosis fugax" OR "angioid streaks" OR "artificial lens" OR "cogan syndrome" OR "color perception" OR "cone dystrophy" OR "duane retraction syndrome" OR "ectopia lentis" OR "epiretinal membrane" OR "familial exudative vitreoretinopathies" OR "fluorescein angiography" OR "leber congenital amaurosis" OR "lens subluxation" OR "limbal stem cell deficiency" OR "meibomian gland" OR "miller fisher syndrome" OR "Mucous Membrane Pemphigoid"  OR "opsoclonus-myoclonus" OR "orbital cellulitis" OR "orbital disease*" OR "orbital myositis" OR "orbital neoplasm*" OR "orbital pseudotumor" OR "persistent hyperplastic primary vitreous" OR "refractive error*" OR "retrobulbar hemorrhage" OR "slit lamp microscopy" OR "stargardt disease" OR "susac syndrome" OR "tolosa-hunt syndrome" OR "vitreous detachment" OR "vitreous hemorrhage" OR "walker-warburg syndrome" OR "weill-marchesani syndrome" OR aberrometry OR amblyopia OR aniridia OR aniseikonia OR anisocoria OR anisometropia OR anophthalmos OR aphakia OR asthenopia OR astigmatism OR blephar* OR blind* OR cataract* OR chalazion OR chorioretinopath* OR choroid* OR coloboma OR cone-rod OR conjunctiv* OR cornea* OR dacryocystitis OR diplopia OR ectropion OR electronystagmography OR electrooculography OR electroretinography OR endophthalmitis OR enophthalmos OR entropion OR exophthalmos OR fluorophotometry OR glaucoma OR gonioscopy OR hemianopsia OR hordeolum OR hydrophthalmos OR hyperopia OR hyphema OR iridocorneal OR iris OR keratiti* OR keratoconus OR lacrimal OR macula* OR microphthalmos OR miosis OR mydriasis OR myopia OR nystagmus OR ocular* OR ophthalmodynamometry OR ophthalmolog* OR  ophthalmopathy OR ophthalmoplegia OR ophthalmoscopy OR optic OR optometry OR  papilledema OR photophobia OR pinguecula OR presbyopia OR pterygium OR pupil OR retina* OR retinitis OR retinoblastoma OR retinopath* OR retinoscopy OR sclera* OR scleritis OR scotoma OR strabismus OR trachoma OR trichiasis OR uveal* OR uveitis OR visual OR vitreoretinopathy OR xerophthalmia)  2. DE "INCLUSIONARY housing programs" OR DE "HOUSING discrimination laws -- United States" OR DE "HOUSING market" OR DE "HOUSING & health" OR DE "HOUSING discrimination laws" OR DE "AFRICAN American homeless persons" OR DE "DISCRIMINATION against the homeless" OR DE "HISPANIC American homeless persons" OR DE "NATIVE American homeless persons" OR DE "TRAMPS" OR DE "ROGUES & vagabonds" OR DE "HOMELESS persons" OR DE "HOMELESS shelters" OR DE "HOMELESS students" OR DE "HOMELESS veterans" OR DE "HOMELESS women" OR DE "RENT" OR DE "LANDLORD-tenant relations" OR DE "TENANTS" OR DE "EVICTION" OR DE "HOUSING" OR DE "CONGREGATE housing" OR DE "COOPERATIVE housing" OR DE "EMERGENCY housing" OR DE "HOME ownership" OR DE "HOMELESSNESS" OR DE "HOUSEHOLDS" OR DE "HOUSING discrimination" OR DE "HOUSING for people with disabilities" OR DE "LOW-income housing" OR DE "MINORITIES -- Housing" OR DE "PUBLIC housing" OR DE "RENTAL housing" OR DE "RURAL housing" OR DE "SHARED housing" OR DE "SLUMS" OR DE "STUDENT housing" OR DE "WOMEN'S shelters" OR DE "HOME ownership" OR DE "HOUSING discrimination" OR DE "PUBLIC housing" OR DE "RENTAL housing" OR DE "HOMEOWNERS" OR DE "HOUSING finance" OR DE "HOUSING laws" OR DE "HOUSING policy" OR DE "HOUSING satisfaction" OR DE "HOME environment" OR DE "DWELLINGS" OR DE "HOUSING" OR DE "HOMELESS families" OR DE "HOMELESS men" OR DE "OLDER homeless persons" OR TI("accessory dwelling unit" OR "displaced people" OR "displaced person*" OR "home moves" OR "residential instabil*" OR "street people" OR apartment* OR condo OR condominium* OR condos OR dweller* OR dwelling OR evicted OR eviction* OR foreclos* OR homeless* OR homeownership OR "homes" OR house OR houses OR housing OR leaseholder* OR rent OR rental OR renter* OR renting OR tenant OR tenants OR townhouse* OR unhoused) OR AB("accessory dwelling unit" OR "displaced people" OR "displaced person*" OR "home moves" OR "residential instabil*" OR "street people" OR apartment* OR condo OR condominium* OR condos OR dweller* OR dwelling OR evicted OR eviction* OR foreclos* OR homeless* OR homeownership OR "homes" OR house OR houses OR housing OR leaseholder* OR rent OR rental OR renter* OR renting OR tenant OR tenants OR townhouse* OR unhoused)  1 AND 2 |
| **Scopus (Elsevier)**  1. TITLE(("aicardi syndrome" OR "amaurosis fugax" OR "angioid streaks" OR "artificial lens" OR "cogan syndrome" OR "color perception" OR "cone dystrophy" OR "duane retraction syndrome" OR "ectopia lentis" OR "epiretinal membrane" OR "familial exudative vitreoretinopathies" OR "fluorescein angiography" OR "leber congenital amaurosis" OR "lens subluxation" OR "limbal stem cell deficiency" OR "meibomian gland" OR "miller fisher syndrome" OR "Mucous Membrane Pemphigoid" OR "opsoclonus-myoclonus" OR "orbital cellulitis" OR "orbital disease" OR "orbital myositis" OR "orbital neoplasm" OR "orbital pseudotumor" OR "persistent hyperplastic primary vitreous" OR "refractive error" OR "retrobulbar hemorrhage" OR "slit lamp microscopy" OR "stargardt disease" OR "susac syndrome" OR "tolosa-hunt syndrome" OR "vitreous detachment" OR "vitreous hemorrhage" OR "walker-warburg syndrome" OR "weill-marchesani syndrome" OR aberrometry OR amblyopia OR aniridia OR aniseikonia OR anisocoria OR anisometropia OR anophthalmos OR aphakia OR asthenopia OR astigmatism OR blephar* OR blind OR cataract* OR chalazion OR chorioretinopath* OR choroid* OR coloboma OR cone-rod OR conjunctiv* OR cornea* OR dacryocystitis OR diplopia OR ectropion OR electronystagmography OR electrooculography OR electroretinography OR endophthalmitis OR enophthalmos OR entropion OR exophthalmos OR fluorophotometry OR glaucoma OR gonioscopy OR hemianopsia OR hordeolum OR hydrophthalmos OR hyperopia OR hyphema OR iridocorneal OR iris OR keratiti* OR keratoconus OR lacrimal OR macula* OR microphthalmos OR miosis OR mydriasis OR myopia OR nystagmus OR ocular* OR ophthalmodynamometry OR ophthalmolog* OR ophthalmopathy OR ophthalmoplegia OR ophthalmoscopy OR optic OR optometry OR papilledema OR photophobia OR pinguecula OR presbyopia OR pterygium OR pupil OR retina* OR retinitis OR retinoblastoma OR retinopath* OR retinoscopy OR sclera* OR scleritis OR scotoma OR strabismus OR trachoma OR trichiasis OR uveal* OR uveitis OR visual OR vitreoretinopathy OR xerophthalmia OR eye OR eyes OR vision) AND ("accessory dwelling unit" OR "displaced people" OR "displaced person" OR "home moves" OR "residential instabil*" OR "street people" OR apartment* OR condo OR condominium* OR condos OR dweller* OR dwelling OR evicted OR eviction* OR foreclos* OR homeless* OR homeownership OR "homes" OR house OR houses OR housing OR leaseholder* OR rent OR rental OR renter* OR renting OR tenant OR tenants OR townhouse* OR unhoused)) |
| **Web of Science (SCI-EXPANDED, SSCI, and ESCI)**  TITLE (("aicardi syndrome" OR "amaurosis fugax" OR "angioid streaks" OR "artificial lens" OR "cogan syndrome" OR "color perception" OR "cone dystrophy" OR "duane retraction syndrome" OR "ectopia lentis" OR "epiretinal membrane" OR "familial exudative vitreoretinopathies" OR "fluorescein angiography" OR "leber congenital amaurosis" OR "lens subluxation" OR "limbal stem cell deficiency" OR "meibomian gland" OR "miller fisher syndrome" OR "Mucous Membrane Pemphigoid" OR "opsoclonus-myoclonus" OR "orbital cellulitis" OR "orbital disease" OR "orbital myositis" OR "orbital neoplasm" OR "orbital pseudotumor" OR "persistent hyperplastic primary vitreous" OR "refractive error" OR "retrobulbar hemorrhage" OR "slit lamp microscopy" OR "stargardt disease" OR "susac syndrome" OR "tolosa-hunt syndrome" OR "vitreous detachment" OR "vitreous hemorrhage" OR "walker-warburg syndrome" OR "weill-marchesani syndrome" OR aberrometry OR amblyopia OR aniridia OR aniseikonia OR anisocoria OR anisometropia OR anophthalmos OR aphakia OR asthenopia OR astigmatism OR blephar* OR blind* OR cataract* OR chalazion OR chorioretinopath* OR choroid* OR coloboma OR cone-rod OR conjunctiv* OR cornea* OR dacryocystitis OR diplopia OR ectropion OR electronystagmography OR electrooculography OR electroretinography OR endophthalmitis OR enophthalmos OR entropion OR exophthalmos OR fluorophotometry OR glaucoma OR gonioscopy OR hemianopsia OR hordeolum OR hydrophthalmos OR hyperopia OR hyphema OR iridocorneal OR iris OR keratiti* OR keratoconus OR lacrimal OR macula* OR microphthalmos OR miosis OR mydriasis OR myopia OR nystagmus OR ocular* OR ophthalmodynamometry OR ophthalmolog* OR ophthalmopathy OR ophthalmoplegia OR ophthalmoscopy OR optic OR optometry OR papilledema OR photophobia OR pinguecula OR presbyopia OR pterygium OR pupil OR retina* OR retinitis OR retinoblastoma OR retinopath* OR retinoscopy OR sclera* OR scleritis OR scotoma OR strabismus OR trachoma OR trichiasis OR uveal* OR uveitis OR visual OR vitreoretinopathy OR xerophthalmia OR eye OR eyes OR vision) AND ("accessory dwelling unit" OR "displaced people" OR "displaced person" OR "home moves" OR "residential instabil*" OR "street people" OR apartment* OR condo OR condominium* OR condos OR dweller* OR dwelling OR evicted OR eviction* OR foreclos* OR homeless* OR homeownership OR "homes" OR house OR houses OR housing OR leaseholder* OR rent OR rental OR renter* OR renting OR tenant OR tenants OR townhouse* OR unhoused)) |
